# CYP3A5 promotes glioblastoma stemness and chemoresistance through fine-tuning NAD^+^/NADH ratio

**DOI:** 10.1186/s13046-024-03254-x

**Published:** 2025-01-03

**Authors:** Wentao Hu, Xiaoteng Cui, Hongyu Liu, Ze Li, Xu Chen, Qixue Wang, Guolu Zhang, Er Wen, Jinxin Lan, Junyi Chen, Jialin Liu, Chunsheng Kang, Ling Chen

**Affiliations:** 1 School of Medicine, Chinese PLA General Hospital, Nankai University, Beijing, China; 2 Department of Neurosurgery, Institute of Neurosurgery of Chinese PLA, Medical School of Chinese PLA, Chinese PLA General Hospital, Beijing, China; 3 Laboratory of Neuro-oncology, Tianjin Neurological Institute, Tianjin Medical University General Hospital, Tianjin, China; 4 Key Laboratory of Post-Neuro Injury Neuro-repair and Regeneration in Central Nervous System, Ministry of Education and Tianjin City, Tianjin, China; 5 China Medical University, Shenyang, Liaoning China

**Keywords:** Glioblastoma stem cell, Chemoresistance, NAD, Mitochondrion, CYP3A5

## Abstract

**Background:**

Glioblastoma multiforme (GBM) exhibits a cellular hierarchy with a subpopulation of stem-like cells known as glioblastoma stem cells (GSCs) that drive tumor growth and contribute to treatment resistance. NAD(H) emerges as a crucial factor influencing GSC maintenance through its involvement in diverse biological processes, including mitochondrial fitness and DNA damage repair. However, how GSCs leverage metabolic adaptation to obtain survival advantage remains elusive.

**Methods:**

A multi-step process of machine learning algorithms was implemented to construct the glioma stemness-related score (GScore). Further in silico and patient tissue analyses validated the predictive ability of the GScore and identified a potential target, CYP3A5. Loss-of-function or gain-of-function genetic experiments were performed to assess the impact of CYP3A5 on the self-renewal and chemoresistance of GSCs both in vitro and in vivo. Mechanistic studies were conducted using nontargeted metabolomics, RNA-seq, seahorse, transmission electron microscopy, immunofluorescence, flow cytometry, ChIP‒qPCR, RT‒qPCR, western blotting, etc. The efficacy of pharmacological inhibitors of CYP3A5 was assessed in vivo.

**Results:**

Based on the proposed GScore, we identify a GSC target CYP3A5, which is highly expressed in GSCs and temozolomide (TMZ)-resistant GBM patients. This elevated expression of CYP3A5 is attributed to transcription factor STAT3 activated by EGFR signaling or TMZ treatment. Depletion of CYP3A5 impairs self-renewal and TMZ resistance of GSCs. Mechanistically, CYP3A5 maintains mitochondrial fitness to promote GSC metabolic adaption through the NAD⁺/NADH-SIRT1-PGC1α axis. Additionally, CYP3A5 enhances the activity of NAD-dependent enzyme PARP to augment DNA damage repair. Treatment with CYP3A5 inhibitor alone or together with TMZ effectively suppresses tumor growth in vivo.

**Conclusion:**

Together, this study suggests that GSCs activate STAT3 to upregulate CYP3A5 to fine-tune NAD⁺/NADH for the enhancement of mitochondrial functions and DNA damage repair, thereby fueling tumor stemness and conferring TMZ resistance, respectively. Thus, CYP3A5 represents a promising target for GBM treatment.

**Supplementary Information:**

The online version contains supplementary material available at 10.1186/s13046-024-03254-x.

## Introduction

Glioblastoma multiforme (GBM) is the most common and lethal malignant brain tumor [1]. Even with the implementation of the standard of care comprising surgery, radiotherapy, and chemotherapy, the current median overall survival for newly diagnosed GBM patients ranges from 15 to 17 months [2]. Glioblastoma stem cells (GSCs), which fuel tumor phenotypic plasticity and heterogeneity, are responsible for tumor initiation, progression, and therapy resistance [3, 4]. GSCs maintain self-renewal through several molecular signaling pathways, such as the Notch pathway and receptor tyrosine kinases [5, 6]. Since temozolomide (TMZ) has become the cornerstone of GBM therapy, an in-depth understanding of the mechanisms underlying TMZ resistance is of paramount importance. O6-methylguanine methyltransferase (MGMT) promoter status, DNA damage repair activity, cell autophagy, temozolomide efflux, etc., have been proposed to be involved in TMZ resistance [7, 8]. Additionally, GSCs have been thought to play a major role in TMZ resistance. TMZ exerts evolutionary selective pressure on GSCs, leading to the proliferation of drug-resistant GSCs characterized by enhanced DNA damage repair capabilities [3, 9]. Nevertheless, the mechanisms through which GSCs maintain self-renewal and derive chemoresistance are still not fully understood.

Metabolic reprogramming is a hallmark of tumors. Although the Warburg effect represents the preference of tumor cells for anaerobic glycolysis, mitochondrial oxidative phosphorylation (OXPHOS) is also indispensable for maintaining the stemness of some cancer stem cells (CSCs). CSCs derived from GBM, pancreatic ductal adenocarcinoma, and leukemia exhibit a dependency on OXPHOS to sustain aggressive proliferation [10–12]. This is attributed to the role of the mitochondrion in supporting tumor metabolic adaptation through energy and various biosynthetic intermediates production [13]. Thus, targeting mitochondrions to disrupt metabolic homeostasis is a promising strategy for GBM treatment.

Nicotinamide adenine dinucleotide (NAD) is a crucial small molecular cofactor in cellular metabolic processes and signaling regulation. Oxidized nicotinamide adenine dinucleotide (NAD⁺) can be converted to reduced nicotinamide adenine dinucleotide (NADH) and vice versa. The NAD⁺/NADH ratio is critical for the activities of several enzymes, including sirtuins (SIRTs) and poly(ADP-ribose) polymerases (PARPs), which are involved in mitochondrial regulation, DNA repair, and stress responses [14, 15]. Malignant cells have a critical demand for NAD⁺ [16, 17]. Recently, it has been found that cancer cells exhibited an elevated ratio of NAD⁺/NADH compared to non-cancerous cells [18]. Moreover, NAD⁺/NADH has been reported to contribute to kidney cancer metastasis and brain tumor-initiating cell immortalization [19, 20]. However, further elucidation is required to uncover the mechanisms by which GSCs regulate and utilize NAD⁺/NADH to obtain advantageous phenotypes.

CYP3A5 is a member of the cytochrome P450 (CYP) enzyme superfamily that almost acts as monooxygenases using NADH or reduced nicotinamide adenine dinucleotide phosphate (NADPH) as electron donors (NAD(P)H + O2 + R → NAD(P)^+^ + RO + H2O) [21]. Thus, CYP enzymes convert NAD(P)H to NAD(P)^+^ in the metabolism of various substances in cells. Various members of the P450 enzyme superfamily have been implicated in tumor progression [22, 23]. CYP3A5 was reported to mediate therapy resistance in pancreatic ductal adenocarcinoma [24, 25]. Nonetheless, the role of CYP3A5 in GBM progression is unknown.

Here, we demonstrate that when stimulated with various factors, GSCs highly express CYP3A5 through STAT3 activation to obtain a survival advantage, including stemness maintenance and drug resistance. This feature of GSCs is attributed to CYP3A5-mediated NAD⁺/NADH elevation, which promotes the activity of SIRT1 and PARP1 to enhance mitochondrial function and DNA damage repair, respectively. Our findings uncover a mechanism where metabolic adaptation confers GSCs survival advantages and highlight targeting CYP3A5 as a potential strategy for GBM treatment.

## Methods

### In silico data acquisition

The Cancer Genome Atlas (TCGA) data were obtained using the TCGAbiolinks R package. Gene Expression Omnibus (GEO) data, including GSE68848, GSE13041, and GSE16011, were downloaded from the website (http://www.ncbi.nlm.nih.gov/geo/). The Chinese Glioma Genome Atlas (CGGA) data were downloaded from the website (http://www.cgga.org.cn/). The single-cell sequencing data for glioblastoma cells were downloaded from the Broad Institute Single-Cell Portal [26].

### Human glioma specimens

The study was conducted in accordance with the Declaration of Helsinki. The tumor and adjacent nontumor tissues used in this study were obtained with written informed consent from patients with gliomas who underwent resection at the Department of Neurosurgery, First Medical Center of Chinese PLA General Hospital. The experimental protocols were approved by the Ethics Committee of the First Medical Center of Chinese PLA General Hospital (No. S2018-089). Tissue microarray slides containing 122 high-grade and 58 low-grade glioma samples were obtained from Shanghai WellBio Technology Co., Ltd.

### Cell culture and reagents

Patient-derived GSCs, which were a gift from Beijing Tiantan Hospital, were used in this study, as previously described [27, 28]. Patient-derived GSCs and U87MG, LN229, and U251 were grown as neurospheres in serum-free media consisting of DMEM/F12, B27 lacking vitamin A (Invitrogen), 2 µg/mL heparin (STEMCELL), 20 ng/mL EGF (R&D), and 20 ng/mL bFGF (R&D). U87-MG, LN229, and U251 cells were purchased from the American Type Culture Collection (ATCC). The spheres were dissociated using Accutase (Sigma) for in vitro experiments. GSCs were cultured in DMEM/F12 supplemented with 10% FBS for 7 days to allow them to differentiate into DGCs. The authenticity of all cultures was monitored via short tandem repeats (STRs), and mycoplasma testing was routinely performed. Nicotinamide mononucleotide, cobicistat, resveratrol, KPT-9247, and WP1066 were purchased from MCE. Temozolomide was purchased from Sigma–Aldrich.

### Xenograft studies

Four-week-old female BALB/c nude mice purchased from Beijing Vital River Laboratory Animal Technology Co., Ltd. were maintained in the pathogen-free barrier animal facility at the Tianjin Neurological Institute of Tianjin Medical University General Hospital. All mouse studies were performed with the approval of the Institutional Animal Ethics and Welfare Committee of Tianjin Medical University (No. IRB2022-DWFL-069). For intracranial tumor xenografts, neurospheres infected with a luciferase lentivirus were stereotactically implanted into the right front lobe of each mouse at a depth of 3 mm. Tumor growth was monitored using the IVIS Lumina imaging station. Mice from different experimental groups were sacrificed on the same day after transplantation to compare the tumor sizes with H&E staining. Then, the brains were harvested, fixed with 4% paraformaldehyde, and subsequently embedded in paraffin and sectioned. For the survival analysis, the animals were maintained until neurological signs were observed. For combination treatment, 5 mg/kg TMZ (Sigma) and 10 mg/kg cobicistat (MCE) were administered via oral gavage at the indicated frequency.

### Immunoblot

Protein was extracted from cells with radioimmunoprecipitation assay (RIPA) protein lysis buffer, separated on SDS‒PAGE gels, and transferred onto polyvinylidene fluoride (PVDF) membranes. After an incubation with primary antibodies at four °C overnight, the membranes were incubated with HRP-conjugated secondary antibodies. The antibodies used for western blotting are listed in Table S1.

### Immunoprecipitation

Equal amounts of 500 µl of cell lysates were incubated with the primary antibody overnight at four °C with rotation. The next day, prewashed magnetic beads were added to the immunocomplex, which was rotated for 30 min at room temperature. The beads were collected via a magnetic separation rack, washed 3 times, boiled in a loading buffer for 10 min at 100 °C, and then subjected to western blot analysis.

### RNA isolation and quantitative real-time PCR

Total RNA was extracted with TRIzol reagent and reverse transcribed. The RT-qPCR assay was subsequently performed using SYBR Green mix on a QuantStudio 3 Real-Time PCR system (Thermo Scientific). GAPDH was used for normalization. The sequences of primers used for RT-qPCR are listed in Table S2.

### Chromatin immunoprecipitation (ChIP) assay

The ChIP assay was performed using the SimpleChIP^®^ Enzymatic Chromatin IP Kit (#9003, CST) according to the manufacturer’s protocol. Briefly, 1 × 10^7^ cells were crosslinked with 1% formaldehyde for 10 min at room temperature and then quenched with glycine. The cells were subsequently collected, and chromatin was digested with micrococcal nuclease, followed by sonication to obtain 150–900 bp DNA fragments. For immunoprecipitation, 5 µl of STAT3 antibody was added to the diluted chromatin and incubated overnight at 4 °C with rotation, followed by the addition of magnetic beads and incubation for 2 h at 4 °C. After the elution of chromatin from magnetic beads and reversal of cross-links, chip DNA samples were purified and analyzed via RT‒qPCR. The primers used are detailed in Table S2.

### Generation of knockout (KO), knockdown (KD), and overexpression cells

Glioma cells were infected with a leti-FLAG-Cas9-Bsdr lentivirus, followed by selection with 10 µg/ml blasticidin to induce stable cas9 expression. Then, CYP3A5 KO cells were generated by transducing leti-sgRNA-puro lentiviral vectors into cas9-expressing cells. Stably transfected cell lines were selected with 2 µg/ml puromycin, and the CRISPR-KO efficiency was evaluated by western blotting. siRNAs targeting human STAT3 and CYP3A5 were transduced into cells using Lipofectamine 3000 (Invitrogen). For CYP3A5 overexpression, cells were transfected with the CYP3A5 overexpression plasmid using Lipofectamine 3000. For EGFRvIII overexpression, cells were transduced with EGFR-vIII lentiviral particles and selected with 2 µg/mL puromycin. The knockdown and overexpression efficiencies were evaluated via RT-qPCR and western blotting. The sequences are provided in Table S3.

### Immunofluorescence staining

Cultures or tissue sections were fixed with 4% formaldehyde for 20 min at room temperature, followed by washes with PBS. The samples were subsequently permeabilized with 0.3% Triton X-100 for 5 min at room temperature. Next, they were incubated with primary antibodies overnight at 4 °C, followed by an incubation with the corresponding secondary antibody for 1 h at room temperature. Finally, the nuclei were counterstained with DAPI.

For TSA staining, after antigen retrieval, the samples were blocked with 3% BSA for 30 min at room temperature and then reacted with primary antibodies at 4 °C overnight, followed by incubation with the appropriate secondary antibodies for 1 h at room temperature. The samples were subsequently incubated with Tyramide signal amplification (TSA) plus a fluorescein kit for 10 min at room temperature. The antibodies were eluted by heating the slides in a microwave. Then, the next primary antibody was added using the steps described above. Nuclei were counterstained with DAPI. All the images were observed with an FV-1000 laser scanning confocal microscope (Olympus Corporation, Japan).

### Immunohistochemistry

The samples were embedded in paraffin. Serial 4 μm sections were cut, deparaffinized, hydrated, and subjected to antigen retrieval. The tissue sections were blocked with goat serum for 1 h at room temperature and then incubated with the appropriate primary antibodies at 4 °C overnight, followed by incubation with a horseradish peroxidase-labeled secondary antibody for 1 h at room temperature. After 3, 3’-diaminobenzidine (DAB) was developed, the sections were covered with coverslips and observed with a microscope. IHC scores were calculated by the software QuPath (Queen’s University, Belfast, Northern Ireland, https://qupath.github.io) using the following formula: [1 × (percent tumor area 1+) + 2 × (percent tumor area 2+) + 3 × (percent tumor area 3+)], where 1+, 2+, or 3 + referred to the intensity of immunohistochemical staining.

### In vitro limiting dilution assay and tumor sphere formation assay

Glioma sphere cells were seeded into 96-well plates at a density of 1, 5, 10, 20, 40, 80, and 160 cells per well, with 10 replicates. After 7 days, wells that did not contain spheres (diameter ≥ 50 μm) were analyzed with the ELDA website (https://bioinf.wehi.edu.au/software/elda/). The tumor sphere formation assay was performed by seeding 1000 glioma sphere cells per well into 96-well plates and culturing them for 7 days. Then, neurospheres ≥ 50 μm in diameter in each well were counted.

### Cell viability assay

The cells were seeded in 96-well plates at a density of 2 or 5 × 10^3^ cells per well for analyses of cell proliferation or drug toxicity, respectively. Cell viability was determined using the CCK-8 test (Dojindo) according to the manufacturer’s instructions. The cell proliferation data were normalized to those on Day 1.

### EdU incorporation assay

The EdU incorporation assay was performed using Click-iT EdU Imaging Kits (Thermo Fisher) according to the manufacturer’s protocol. Briefly, GSC spheres were incubated with 10 µM EdU for 2 h. The cells were subsequently fixed and permeabilized, followed by the addition of a Click-iT reaction cocktail and Hoechst 33,342 staining. The proliferation index was calculated by dividing the number of EdU-labeled cells by the number of Hoechst-labeled cells, which was conducted by a blinded investigator.

### Mitochondrial DNA (mtDNA) quantification

Total DNA was extracted from the cell line using QIAamp DNA mini kits (Qiagen) according to the manufacturer’s protocol. Then, the nuclear and mitochondrial DNA contents were detected via RT-qPCR. Relative mtDNA quantification was conducted by normalizing the abundance of mitochondrial DNA (D-LOOP and MT-CO2) to that of nuclear DNA (beta-2-microglobulin, B2M). The primers used are listed in Table S2.

### Seahorse extracellular flux assay

The oxygen consumption rate (OCR) was detected using an XFe24 Extracellular Flux Analyzer (Agilent) according to the manufacturer’s instructions. Briefly, the cells were plated into Seahorse Bioscience culture plates in Seahorse XF RPMI medium buffer. Then, mitochondrial function was assessed by sequentially injecting 2 µM oligomycin, 2 µM FCCP, and 0.5 µM rotenone/antimycin A. The OCR data were normalized to the number of cells. Data were analyzed by the Seahorse XFe24 Wave software.

### Transmission electron microscopy

The cells were collected and fixed with 2.5% glutaraldehyde at 4 °C. After pre-embedding in agarose, the cells were fixed with 1% osmium tetroxide and dehydrated with increasing ethanol concentrations. Resin blocks were prepared by embedding cells in EMBed 812 medium followed by a polymerization process. Before imaging, ultrathin sections were prepared using an ultramicrotome and stained with uranyl acetate and lead citrate. Images were captured with a transmission electron microscope (HITACHI) and analyzed with ImageJ software.

### Transcriptomics

Total RNA was extracted using TRIzol reagent (Invitrogen) according to the manufacturer’s protocol. The library was subsequently constructed following an RNA integrity assessment and quantification. RNA sequencing was performed using the Illumina NovaSeq 6000 platform by OE Biotech Co., Ltd. (Shanghai, China). Clean data were obtained from raw reads via fastp and subsequently mapped to the reference genome using HISAT2. DESeq2 was used to perform the differential expression analysis. Gene set enrichment analysis (GSEA) was performed using the clusterProfiler R package, and the results were visualized using the Enrichplot R package. Mitochondrial pathway gene sets were obtained from MitoCarta 3.0 (Broad Institute).

### Untargeted metabolomics

The cell samples were collected and resuspended in prechilled 80% methanol. The thawed samples were sonicated and centrifuged to obtain the supernatant, which was then freeze-dried and dissolved in 10% methanol for LC-MS/MS. Ultrahigh-performance liquid chromatography-tandem mass spectrometry (UHPLC‒MS/MS) analyses were conducted utilizing a Vanquish UHPLC system (Thermo Fisher Scientific, Germany) interfaced with an Orbitrap Q Exactive TMHF mass spectrometer (Thermo Fisher Scientific, Germany) at Novogene Co., Ltd. (Beijing, China). The raw data were analyzed with Compound Discoverer 3.3 (CD3.3, Thermo Fisher Scientific) software. Metabolite identification was conducted through the mzCloud, mzVault, and MassList databases.

### Detection of NAD+/NADH levels

The intracellular NAD^+^/NADH ratio was assessed using a WST-8 NAD^+^/NADH assay kit (Beyotime) according to the manufacturer’s instructions. Briefly, 1 × 10^6^ cells were collected and lysed in 200 µl of ice-cold lysis buffer, followed by centrifugation at 12,000 × g for 10 min at 4 °C. A portion of the supernatant was incubated at 60 °C for 30 min to selectively degrade NAD^+^ to measure the level of NADH. The ratio of NAD^+^/NADH was subsequently measured with a microplate reader at 450 nm.

### ATP measurement

In this study, mitochondrial ATP production levels were quantified by seeding 5 × 10^5^ cells into individual wells of a 6-well plate. The cells were subsequently incubated in a medium supplemented with 2 µM oligomycin (MCE) to selectively block mitochondrial oxidative ATP production for 2 h. ATP levels were measured using an ATP Assay Kit (Beyotime) according to the manufacturer’s instructions. Mitochondrial ATP production was derived by subtracting the amount of ATP in cells treated with oligomycin from that in untreated cells. The ATP levels were normalized to the protein concentration of each sample, which was determined by the bicinchoninic acid protein assay.

### Mitochondrial activity assessment

Mitochondrial activity was measured using MitoTracker Deep Red (MDR; Thermo Fisher Scientific) and JC-1 Kit (Beyotime). Briefly, the cells were incubated with MDR or JC-1 for 20 min at 37 °C, followed by a flow cytometry analysis. Alternatively, GSCs were immobilized on coverslips precoated with matrix gel and subsequently incubated with MDR and Hoechst for 30 min at 37 °C, followed by a fluorescence microscopy analysis.

### Flow cytometry analysis of apoptosis and the cell cycle

The apoptosis assay was conducted with 7-AAD and Annexin V according to the manufacturer’s instructions. A flow cytometer (BD Biosciences) was used to detect the fraction of apoptotic cells. For cell cycle analysis, the cells were harvested and preserved in 70% ethanol for 12 h at 4 °C. The fixed cells were subsequently rinsed with PBS and subjected to RNase I treatment. Finally, the cells were subjected to PI staining for 15 min before the cell cycle analysis using a BD flow cytometer.

### Synergy calculations

The SynergyFinder web interface was used to assess the synergy of the combined drugs [29]. The highest single agent (HSA) model was employed to compute synergy scores, where a score exceeding 10 signified a synergistic interaction.

### Liquid chromatography-mass spectrometry (LC-MS)

The tumor-bearing mice were administered with cobicistat (10 mg/kg) dissolved in solution buffer by gavage. At predosing (0 h) and 0.5, 1, 4, 8, and 12 h postdosing, blood plasma, brain, and xenograft tissues were collected and analyzed using a Waters UPLC System. The internal standard curve and the sample dilution factor were used to calculate the cobicistat concentration in the samples, and their levels were determined by measuring the area under the curve and maximum concentration.

### Bioinformatics analysis

The weighted gene coexpression network analysis (WGCNA) was conducted with the R package “WGCNA”. The least absolute shrinkage and selection operator (LASSO) regression was performed using the R package “glmnet”. Nonconsensus clustering was conducted using the R package “ConsensusClusterPlus”. The survival analysis was conducted via the “survival”, “survminer”, “timeROC”, and “rms” R packages. The functional enrichment analysis was performed using the R packages “clusterProfiler” and “GSVA”. Tumor immune cell infiltration levels were assessed with CIBERSORTx. The enrichment scores of the immune exclusion-related gene sets were calculated using the “IOBR” R package. The tracking tumor immunophenotype (TIP) analysis was performed using an online tool (http://biocc.hrbmu.edu.cn/TIP/index.jsp).

### Construction of the glioma stemness-related score (GScore)

We first calculated the mRNA expression- and DNA methylation-based stemness indices (mRNAsi and mDNAsi) of TCGA glioma samples using a one-class logistic regression (OCLR) model trained on pluripotent stem cells and their differentiated progenitors [30]. In contrast to mRNAsi, elevated mDNAsi levels were significantly correlated with a worse glioma prognosis, increased stemness properties, and the enrichment of cancer malignancy-associated gene sets. Thus, we adopted mDNAsi to measure glioma stemness features. Nevertheless, the implementation of mDNAsi is resource-intensive and inconvenient, necessitating the availability of patients’ DNA methylation sequencing data. Therefore, we construct a scoring system by leveraging the expression of a select few genes to delineate glioma stemness levels through a multistep process of machine learning algorithms. By performing a WGCNA to construct a scale-free coexpression network, the turquoise module was identified as exhibiting the highest correlation with the mDNAsi. Then, 260 key candidate genes in the turquoise module with a module membership > 0.7 and a gene significance > 0.5 were screened. Subsequently, 260 candidate genes were subjected to least absolute shrinkage and selection operator (LASSO)–Cox regression analysis, which was integrated with 10-fold cross-validation and 1000 iterations. Seven hub genes (CLIC1, GUSB, MSN, NTAN1, TGIF1, DCTD, and GLA) with nonzero regression coefficients were then obtained to construct the GScore (Table S4). The GScore was constructed from the mRNA expression of seven hub GSC-related genes weighted by the multivariate Cox regression coefficient: GScore = $\:\sum\:Coefficient\left(mRNAi\right)*Expression\left(mRNAi\right)$. Consensus clustering was implemented to determine the number of clusters using these candidate genes. The optimal number of clusters for TCGA glioma or CGGA glioma samples was 2 according to a comprehensive consideration of the area under the cumulative distribution function (CDF) curves, the proportion of ambiguous clustering (PAC) algorithm, consensus heatmaps, and Kaplan–Meier curves. Thus, the GScore categorized patients into a high-score group (Z score ≥ 0) and a low-score group (Z score < 0).

### Statistical analysis

All the statistical analyses were performed using R (version 4.1.3) and GraphPad Prism (version 9.5.0). Two-tailed unpaired Student’s t-tests and Wilcoxon tests were adopted to compare the differences between two sets of continuous variables. One-way analysis of variance was applied for multiple groups of data. For the survival analysis, Kaplan‒Meier survival curves were analyzed using log-rank statistics. All the data are presented as the means ± SDs unless specified otherwise. *P* values ≤ 0.05 were considered significant.

## Results

### GScore reflects glioma stemness status and clinical outcomes

**Fig. 1:**
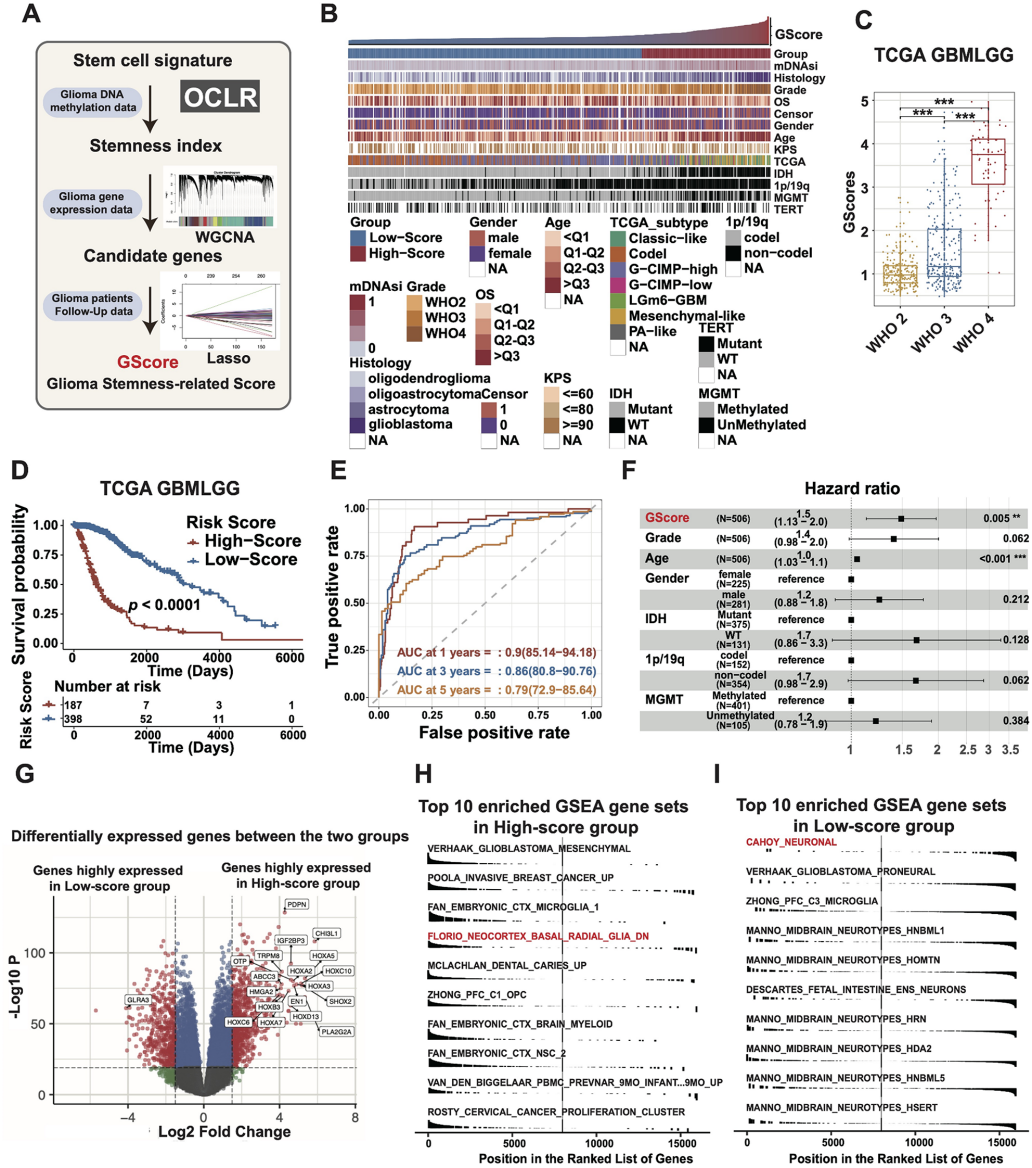
GScore reflects glioma stemness status and clinical outcomes. **(A)** The flowchart describing the strategy of construction of glioma stemness-related score (GScore). **(B)** The landscape of GScore among clinical and molecular factors in TCGA glioma cohort. Columns represent LGG and GBM samples sorted by GScore from low to high. **(C)** The GScore by different WHO grades of TCGA glioma cohort. *p*-values obtained by the One-way ANOVA test. **(D)** The Kaplan–Meier curves of TCGA glioma samples stratified by GScore. *p*-value obtained by the Log-rank test. **(E)** Time-dependent ROC curve at 1-, 3- and 5-years of OS for GScore in the TCGA glioma cohort. **(F)** The forest plot of multivariate Cox regression analysis showing GScore is an independent predictive factor. **(G)** The volcano plot of differentially expressed genes (DEGs) between high-GScore and low-GScore groups. **(H-I)** Gene Set Enrichment Analysis (GSEA) of the top 10 enriched pathways in the high-GScore group and low-GScore group, which was evaluated in the context of all gene sets from the MSigDb Database. Significant results are presented as, ns *p* > 0.05, *p < 0.05, **p < 0.01, ***p < 0.001, ****p < 0.0001

We developed a machine learning-based glioma stemness-related score (GScore) to assess the stemness status of glioma patients conveniently and specifically (Fig. 1A, S1A-R). Next, we investigated the relationship between the GScore and several key clinical and molecular characteristics among glioma patients (Fig. 1B). As GScore increases, glioma patients tend to have higher WHO grades and shorter overall survival (OS) and are more likely to exhibit isocitrate dehydrogenase (IDH) wildtype status and MGMT promoter unmethylation, which all indicate that elevated GScore is associated with poor clinical outcomes (Fig. 1B). Statistically, patients with higher GScore generally have higher WHO grade and shorter survival than those with lower score (Fig. 1C-D). The GScore exhibits robust predictive performance in assessing the prognosis of glioma patients, as indicated by ROC curves (Fig. 1E). The multivariate Cox regression analysis revealed that the GScore was an independent and strong factor for predicting the glioma prognosis (Fig. 1F). Furthermore, independent covariates obtained after multivariate Cox regression were used to construct an OS prediction model visualized in a pictorial nomogram (Fig. S2A-B). Consistently, significant differences in OS between the high-score and low-score groups were observed in multiple glioma testing datasets (Fig. S2C-H). The gene mutation event analysis revealed that mutations in the TP53 gene (36%) were most common in the high-score group, followed by IDH1 (24%), EGFR (22%), and PTEN (22%) (Fig. S2I). Altogether, these results indicate that glioma stemness index-derived GScore predicts clinical outcomes for glioma patients.

Next, to test whether GScore indeed reflects glioma stemness status, an analysis of differentially expressed genes between the two groups was performed, followed by a gene set pathway enrichment analysis (Fig. 1G). The top 10 enriched pathways in both the high-score and the low-score groups were identified through gene set enrichment analysis (GSEA) using all the gene lists from the MSigDB database (Table S5). As expected, in contrast to the low-score group, the top 10 enriched pathways in the high-score group are mainly composed of pathways of brain stem cells and malignancy (Fig. 1H). Conversely, the top 10 enriched pathways of the low-score group mainly comprise pathways of well-differentiated brain cells (Fig. 1I). These data indicate that GScore indeed has a good performance to reflect the tumor stemness status of glioma patients. To further expand the clinical applicability of the GScore, we wonder whether GScore could also predict the immune status of glioma patients in light of the close relationship between tumor stemness and tumor suppressive immunity. Indeed, single-sample gene set enrichment analysis (ssGSEA) reveals that the immune exclusion signature is elevated in the high-score group, accompanied by an increased abundance of immunosuppressive immune cells (Fig. S2J-L). Overall, the GScore demonstrates a promising reflection of glioma stemness while also offering insights into the clinical outcomes and immune microenvironment status of glioma patients.

### The identified CYP3A5 target is upregulated in GSCs and correlates to GBM progression

**Fig. 2:**
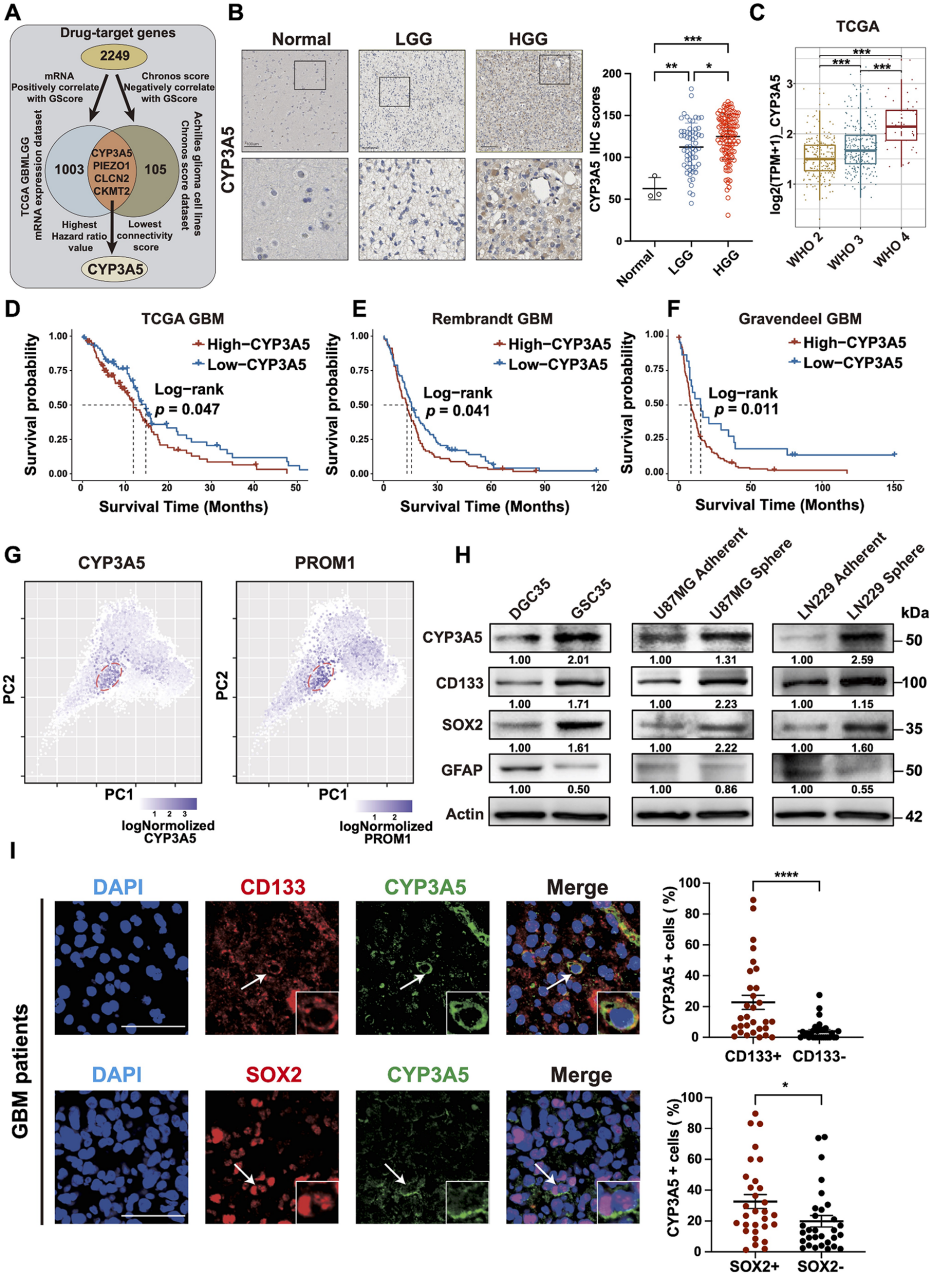
CYP3A5 target is associated with GBM progression and upregulated in GSCs. **(A)** The flowchart describing the strategy of identifying the CYP3A5 target. **(B)** Representative immunohistochemistry images and quantification of CYP3A5 expression measured on a microarray that contains low-grade glioma (LGG, *n* = 59) and high-grade glioma (HGG, *n* = 121), and 3 normal brain tissues. scale bar, 100 μm. *p*-values obtained by one-way ANOVA. **(C)** The boxplot of CYP3A5 expression levels in individual samples of TCGA glioma cohort stratified by WHO grades. *p*-values obtained by one-way ANOVA. **(D–F)** The Kaplan–Meier curves of GBM samples stratified by the optimal cut-off value of CYP3A5 expression level from various datasets. *p*-values obtained by Log-rank test. **(G)** Principal component analysis (PCA) plots of single-cell RNA-sequencing data of 65,655 GSCs and 14,207 GBM cells. Each dot denotes a cell sample. Color represents the mRNA expression level of CYP3A5 or PROM1. **(H)** Western blot analysis of CYP3A5, CD133, SOX2, and GFAP in GSC spheres and their matched adherent DGCs. **(I)** Immunofluorescence analysis of CYP3A5, SOX2, and CD133 in five randomly selected microscope fields from 6 GBM samples (*n* = 30); scale bars, 50 μm. *p*-values obtained by the Mann-Whitney U test. Error bars represent mean ± SD. Significant results are presented as, ns *p* > 0.05, *p < 0.05, **p < 0.01, ***p < 0.001, ****p < 0.0001

To identify druggable target genes with potential therapeutic implications for patients exhibiting high stemness status, a total of 2249 drug-target genes were included for selection. These genes were derived after the duplication removal of more than 6,000 compounds retrieved from the Drug Repurposing Hub created by Broad Institute, which aims to take thousands of drugs already approved to safely treat diseases or potential drugs to find new uses for these old medicines (Fig. 2A).

First, among these drug-target genes, glioma stemness-related genes were identified with gene expression levels positively correlated to GScore (correlation coefficient > 0.2, Fig. S3A). Subsequently, we calculated the GScore for each glioma cell line from the CCLE project and retrieved the Chronos score of these cell lines from the DepMap dataset. Chronos scores represent the importance of a gene of interest for a given cell line through cell depletion assay, a lower Chronos score of the gene denotes a more essential role in cell fitness. Therefore, glioma-dependent genes were identified with Chronos score inversely associated with the GScore (correlation coefficient < -0.5, Fig. S3B). Finally, four potential therapeutic drug targets —CKMT2, CYP3A5, CLCN2, and PIEZO1—emerged by combining both analyses above (Fig. 2A). Among four candidates, CYP3A5 expression is associated with the poorest prognostic outcomes according to a single covariate Cox proportional hazards model in glioma patients (Fig. S3C). Moreover, we quired the Cmap database to assess the perturbation effect of gene depletion on tumor stemness status. Connectivity scores obtained from this analysis mean that a lower score signifies a stronger ability of target depletion to disrupt the tumor’s maintenance of stem-like properties in this context. In the glioma cell line, we found that CYP3A5 depletion had the lowest normalized connectivity score, suggesting the strongest ability to reverse the high GScore signature for glioma (Fig. S3D). Collectively, our findings suggest that CYP3A5 may serve as a promising druggable target for glioma patients with high stemness status.

The general expression status of CYP3A5 was subsequently evaluated in association with various clinical and molecular characteristics among glioma patients (Fig. S3E). Notably, CYP3A5 expression escalates with increasing the WHO grades of glioma, as supported by the results of immunohistochemical (IHC) staining of the glioma tissue microarray, as well as the mRNA expression data from TCGA glioma and glioma samples of our center (Fig. 2B-C, S3F). Additionally, CYP3A5 expression is higher in highly aggressive DNA methylation-based glioma subtypes (Fig. S3G). Furthermore, a significant association was identified between elevated expression of CYP3A5 and an unfavorable prognosis for GBM patients, as tested in multiple databases (Fig. 2D-F). Together, these results indicate that CYP3A5 notably correlates to GBM progression.

Next, we tested whether CYP3A5 was involved in GBM stemness. We found that the CYP3A5 expression level was positively correlated with the expression of most stemness-related genes, as supported by the analysis of bulk transcriptomic data of gliomas in TCGA and CGGA cohorts (Fig. S3H). Moreover, principal component analysis of single-cell RNA-sequencing data of 65,655 GSCs and 14,207 GBM cells revealed that the area where CYP3A5 was highly expressed harbored cells that also markedly expressed cancer stem cell markers, such as PROM1, SOX2, EZH2 and NOTCH1 (Fig. 2G, S3I) [26]. Furthermore, CYP3A5 was upregulated in GSCs maintained under serum-free conditions compared with differentiated glioblastoma adherent cells (DGCs) cultured in serum-supplemented medium (Fig. 2H). Additionally, our investigation revealed the preferential expression of CYP3A5 in cancer cells expressing the GSC markers SOX2 and CD133 via co-immunofluorescence (co-IF) staining of human GBM surgical specimens (Fig. 2I). Together, these results imply that CYP3A5 is preferentially expressed by GSCs.

Next, we investigated the potential impact of CYP3A5 on mesenchymal GSCs, given our previous analysis primarily focused on proneural GSC markers. We began by performing a differential expression analysis of CYP3A5 across various GBM subtypes, which revealed comparable expression levels of CYP3A5 between mesenchymal and proneural GBM patients (Fig. S4A). Moreover, similar to the aforementioned proneural markers, CYP3A5 is highly expressed in GSCs that also exhibit mesenchymal markers such as BMI1 and CD44, supported by both single-cell RNA sequencing data and co-IF analysis of GBM samples (Fig. S4B, C). Furthermore, knocking down CYP3A5 expression in the patient-derived mesenchymal GSC line BNI-20-1-S, which has been previously reported [28], resulted in a reduction of self-renewal capability (Fig. S4D, E). These analyses suggest a potential uniform role of CYP3A5 across both proneural and mesenchymal GSCs.

Collectively, these results demonstrate that CYP3A5 upregulated in GSCs is associated with GBM progression, indicative of CYP3A5 as a potential GBM therapeutic target.

### CYP3A5 deficiency impairs GSC maintenance

**Fig. 3:**
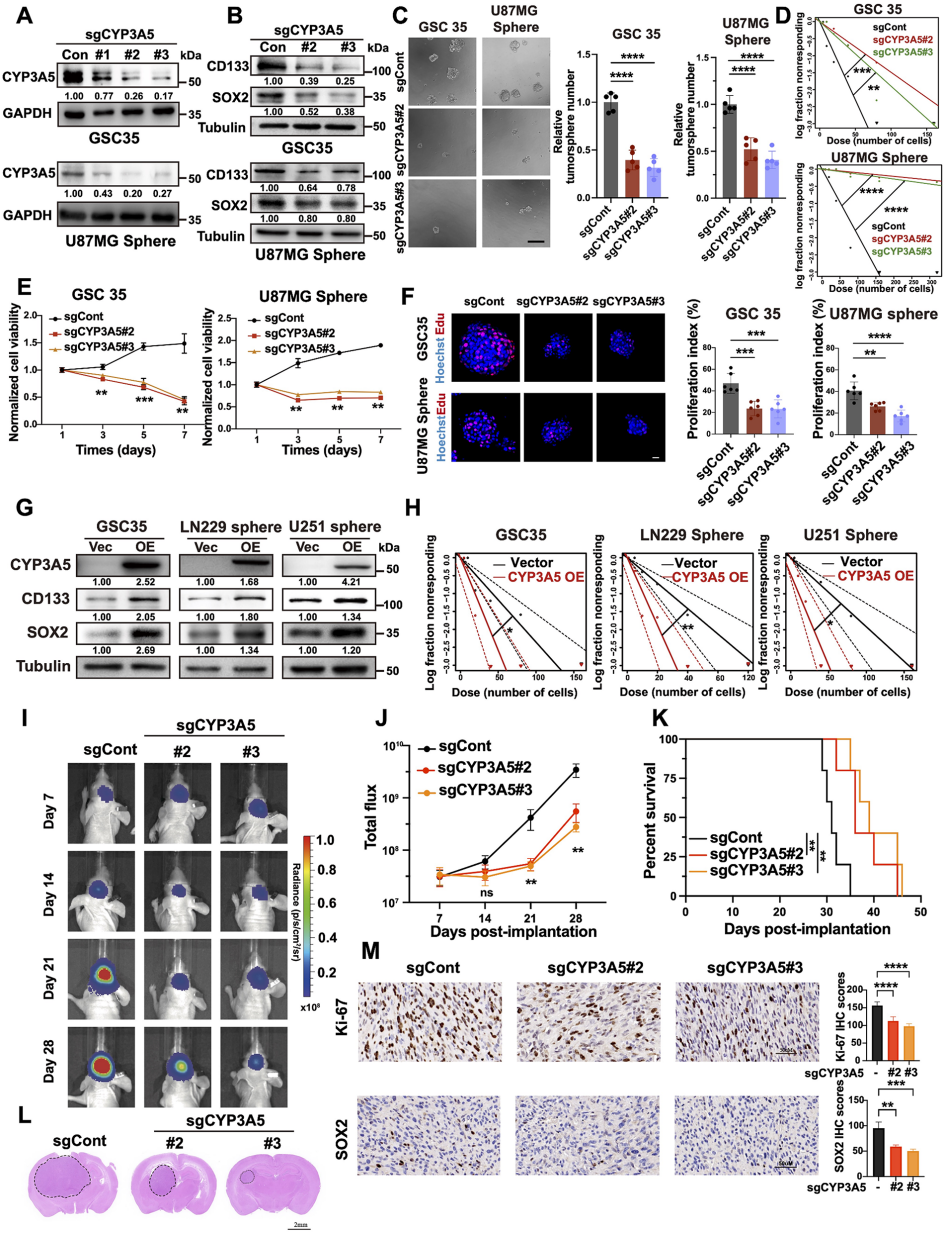
CYP3A5 sustains GSC self-renewal. **(A)** Immunoblots for CYP3A5 in GSCs with or without CYP3A5 KO. **(B)** Immunoblots showing CYP3A5 KO decreased the protein levels of SOX2 and CD133 in GSCs. **(C)** Representative images and quantification of neurospheres in GSCs with or without CYP3A5 KO (*n* = 5). *p*-values obtained by one-way ANOVA; Scale bar, 200 μm. **(D)** Extreme limiting dilution assays (ELDAs) showing CYP3A5 KO diminished the frequency of neurosphere formation. **(E)** Cell viability assay showing CYP3A5 KO impeded GSCs growth (*n* = 3). *p*-values obtained by one-way ANOVA. **(F)** Representative images and quantification of proliferation index (EdU + cells/Hoechst cells) of wild-type and CYP3A5-KO GSCs (*n* = 6). *p*-values obtained by one-way ANOVA. Scale bar, 20 μm. **(G)** Immunoblots of CYP3A5, SOX2, and CD133 in GSCs with or without CYP3A5 overexpression. **(H)** ELDAs show the enhanced ability of neurosphere formation after CYP3A5 overexpression in GSCs. **(I-J)** Representative images and quantification of bioluminescence intensities of nude mice intracranially implanted with U87MG GSCs with or without CYP3A5 KO (*n* = 5). *p*-values obtained by one-way ANOVA. **(K)** Kaplan-Meier survival curves of the nude mice (*n* = 5). *p*-values obtained by Log-rank test. **(L)** Representative images of H&E staining of tumor sections. Scale bar, 2 mm. **(M)** Representative images and quantification of immunohistochemistry staining of Ki-67 and SOX2 (*n* = 5). *p*-values obtained by one-way ANOVA. Scale bars, 50 μm. Error bars represent mean ± SD. Significant results are presented as, ns *p* > 0.05, *p < 0.05, **p < 0.01, ***p < 0.001, ****p < 0.0001

To further probe the role of CYP3A5 in GSCs maintenance, we knocked out or overexpressed CYP3A5 in GSCs according to the endogenous expression of CYP3A5 (Fig. S5A). The targeted KO of CYP3A5 using two nonoverlapping sgRNAs resulted in the efficient deletion of endogenous CYP3A5 expression and a notable reduction in the expression levels of the GSC markers CD133 and SOX2 (Fig. 3A-B). GSC self-renewal was also suppressed upon CYP3A5 KO, as supported by the results of the sphere formation assay and in vitro limiting dilution assay (Fig. 3C-D). Moreover, the attenuation of the proliferation capacity of GSCs following CYP3A5 KO was observed in both the cell viability assay and the EdU incorporation assay (Fig. 3E-F). Consistently, an increase in the expression of the GSC markers CD133 and SOX2 and an increase in GSC self-renewal were also detected following ectopic CYP3A5 expression in GSCs (Fig. 3G-H). Upon CYP3A5 KO, significant GSC cell cycle arrest characterized by the accumulation of cells in the sub-G0 and G1 phases and the downregulation of cell cycle markers was observed, rather than the occurrence of obvious apoptotic cell death (Fig. S5B-D). Notably, no significant changes in the cell proliferation capacity of DGCs were observed with CYP3A5 KO (Fig. S5E), indicating a role for CYP3A5 in fueling the propagation of GSC rather than DGCs. Altogether, these results suggest that CYP3A5 sustains the self-renewal of GSCs.

To further probe whether CYP3A5 deficiency would impede GBM growth, we established orthotopic GBM xenografts in nude mice. We found that CYP3A5 KO in GSCs impeded xenograft tumor growth, as evidenced by bioluminescence imaging analyses (Fig. 3I-J). Moreover, CYP3A5 KO extended mouse survival compared to CYP3A5 WT (Fig. 3K). Hematoxylin and eosin (H&E) staining of tumors revealed that tumor sizes were smaller in mice implanted with CYP3A5 KO GSCs (Fig. 3L). In addition, CYP3A5 KO led to a significant reduction in the number of SOX2 + and Ki67 + tumor cells within GBM xenografts (Fig. 3M). Collectively, these results indicate an important role of CYP3A5 in GSCs maintenance and GBM growth.

### CYP3A5 modulates the NAD+/NADH ratio to promote self-renewal and TMZ resistance of GSCs

**Fig. 4:**
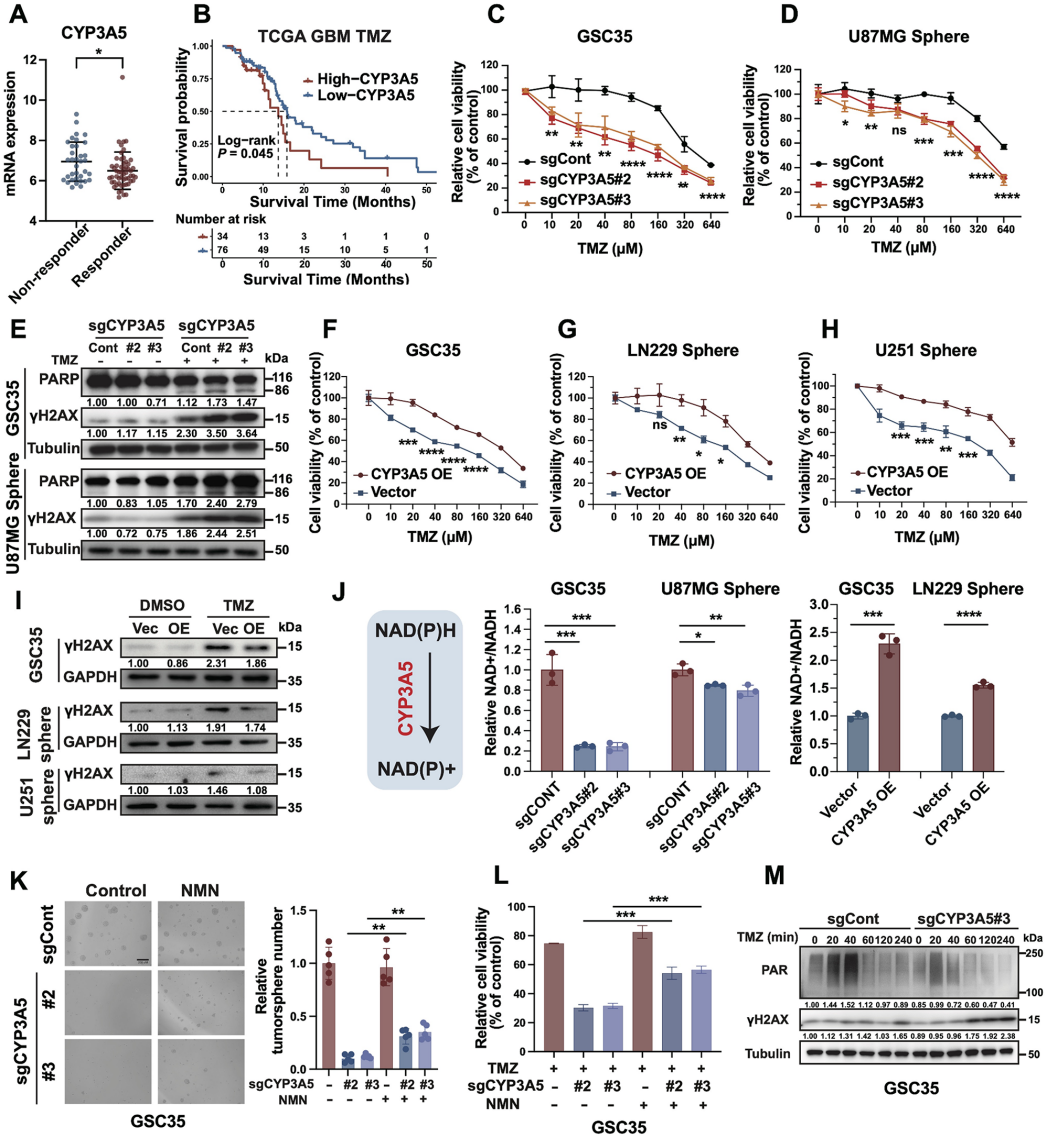
CYP3A5 modulates the NAD^+^/NADH ratio to promote self-renewal and TMZ resistance of GSCs. **(A)** The Box plot showing the mRNA expression of CYP3A5 in GBM patients following TMZ treatment from ROC plotter datasets (Responders *n* = 55, Non-responders *n* = 35). *p*-value obtained by the Mann-Whitney U test. **(B)** The Kaplan–Meier curves of TCGA GBM samples treated with TMZ stratified by the optimal cut-off value of CYP3A5 expression level. *p*-value obtained by the Log-rank test. **(C-D)** Cell viability assays with increasing concentrations of TMZ in GSCs with or without CYP3A5 KO (*n* = 3); *p*-values obtained by one-way ANOVA. **(E)** Immunoblots of cleaved PARP and γH2AX in control and CYP3A5 KO GSCs treated with DMSO or TMZ. **(F–H)** Cell viability assays with increasing concentrations of TMZ in GSCs with or without CYP3A5 overexpression (*n* = 3); *p*-values obtained by t-test. **(I)** Immunoblot of γH2AX in control and CYP3A5 overexpression GSCs treated with DMSO or TMZ. **(J)** Alterations in NAD-to-NADH ratio of CYP3A5-KO or CYP3A5-overexpressing GSCs (*n* = 3). *p*-values obtained by one-way ANOVA and t-test. **(K)** Representative images and quantification of neurosphere formation in control and CYP3A5 KO GSCs treated with vehicle or NMN (*n* = 5); *p*-values obtained by the Mann-Whitney U test; Scale bar, 200 μm. **(L)** Relative cell viability post TMZ treatment in control and CYP3A5 KO GSCs treated with vehicle or NMN (*n* = 3). *p*-values obtained by the t-test. **(M)** Immunoblots of poly ADP-ribosylation (PAR) and γH2AX in control and CYP3A5-KO GSCs treated with TMZ with various time points. Error bars represent mean ± SD. Significant results are presented as, ns *p* > 0.05, *p < 0.05, **p < 0.01, ***p < 0.001, ****p < 0.0001

Given that GSCs are widely acknowledged as the primary culprits behind therapy resistance, we wondered whether CYP3A5 also promoted GSC chemoresistance [31]. Among GBM patients receiving TMZ, higher levels of CYP3A5 were observed in nonresponsive patients compared to their responsive counterparts in ROC plotter datasets (Fig. 4A). Furthermore, among GBM patients receiving TMZ, those with low CYP3A5 expression had a better prognosis than those with high expression (Fig. 4B). Next, we tested whether CYP3A5 promoted the resistance of GSCs to TMZ by cell viability assays where cells were treated with increasing doses of TMZ. We discovered that CYP3A5 KO conferred GSCs more sensitive to TMZ treatment (Fig. 4C-D). Furthermore, CYP3A5 KO augmented TMZ-induced DNA damage and cell apoptosis, as indicated by elevated levels of γH2AX and cleaved PARP (Fig. 4E). Consistently, overexpression of CYP3A5 enhanced the resistance of GSCs to TMZ, as indicated by increased cell viability and reduced γH2AX levels after TMZ treatment (Fig. 4F-I). Altogether, these results indicate that CYP3A5 promotes GSC resistance to TMZ by mitigating DNA damage.

Next, we investigated mechanisms underlying the observed increase in TMZ resistance and self-renewal by CYP3A5 in GSCs. Given that CYP3A5 is a member of the cytochrome P450 enzyme family that catalyzes the oxidation of substrates by converting NADH to NAD^+^, we hypothesized that CYP3A5 might mediate GSC maintenance and chemoresistance by modulating the NAD^+^/NADH level [21]. To this end, alterations in the NAD^+^/NADH ratio were detected following CYP3A5 KO or overexpression. We noted that CYP3A5 KO decreased the NAD^+^/NADH ratio of GSCs (Fig. 4J). Meanwhile, significant elevations of the ratio were observed when overexpressing CYP3A5 (Fig. 4J). Additionally, nontargeted metabolomics of GSCs with CYP3A5 KO also showed that CYP3A5 KO led to a decrease in the NAD^+^/NADH ratio (see below). These results suggest that CYP3A5 fine-tunes the NAD^+^/NADH ratio in GSCs. Then, we supplemented CYP3A5-KO GSCs with nicotinamide mononucleotide (NMN), which has been shown to be able to increase the ratio of NAD^+^/NADH [32]. We found that the replenishment of NMN alleviated the inhibitory effect of CYP3A5 KO on GSC self-renewal (Fig. 4K). NMN replenishment also partially restored TMZ resistance in CYP3A5-KO GSCs (Fig. 4L). Next, we used KPT-9247, a specific inhibitor of nicotinamide phosphoribosyltransferase (NAMPT), to decrease the total level of NAD. The use of KPT-9247 led to the mitigation of the facilitating effect of CYP3A5 overexpression on the neurosphere number (Fig. S5F). Altogether, these results suggest that CYP3A5 mediates GSCs proliferation and TMZ resistance through modulating NAD^+^/NADH levels.

Given that NAD^+^ is critical for the synthesis of poly(ADP‒ribosyl)ation (PAR) by PARPs for DNA damage repair [14], we wondered if the levels of PAR post-TMZ treatment would be influenced by CYP3A5 KO. Strikingly, upon CYP3A5 KO, PAR levels measured after TMZ treatment were significantly decreased accompanied by elevated DNA damage marker γH2AX, suggesting that CYP3A5 deficiency replicated the effect of decreased NAD^+^ on PARP activity. (Fig. 4M). Collectively, these results demonstrate that CYP3A5 impacts the activity of NAD^+^ dependent-PARP to regulate the DNA repair capacity of GSCs.

### CYP3A5 fine-tunes the mitochondrial fitness required for the metabolic adaptation of GSCs

**Fig. 5:**
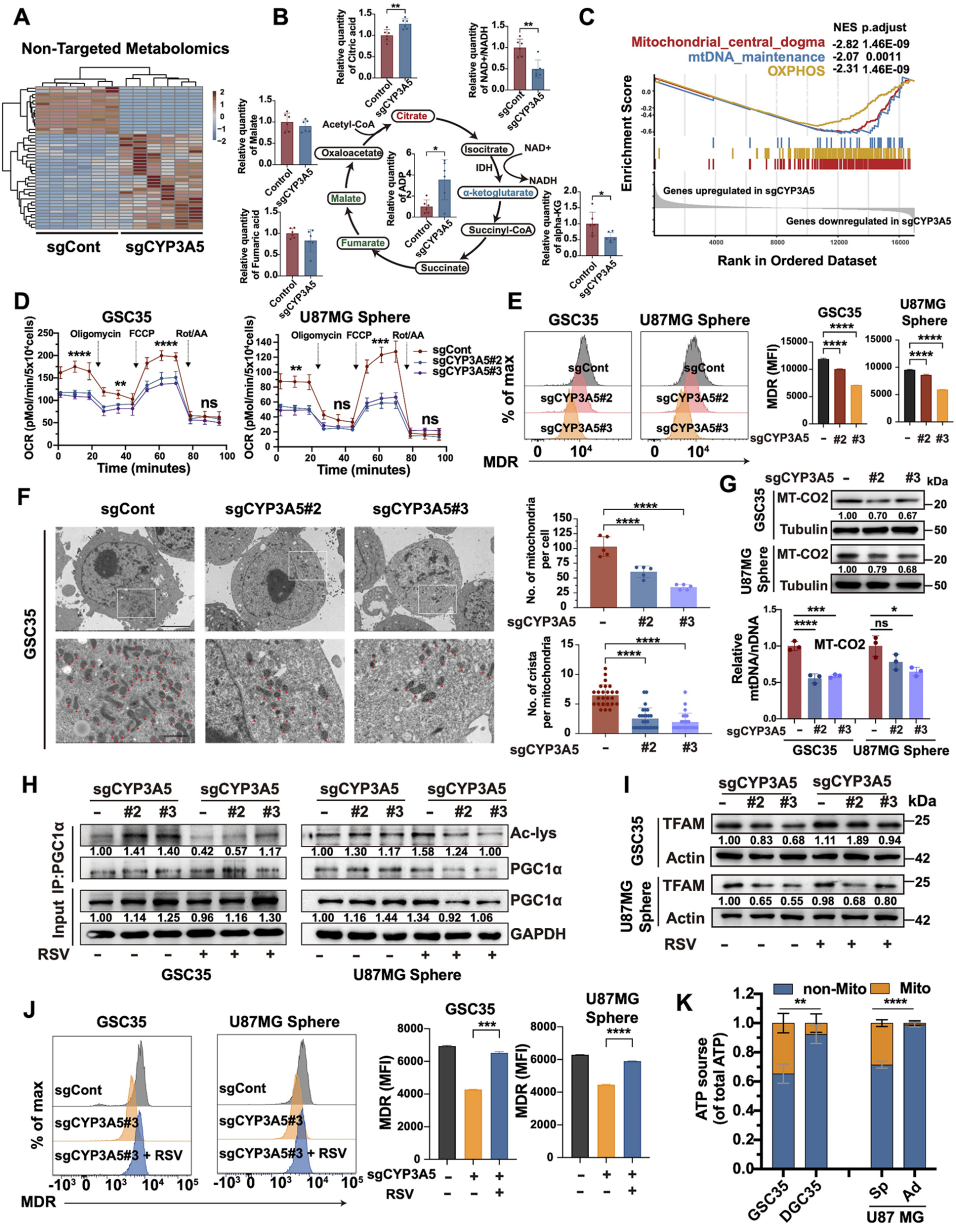
CYP3A5 fine-tunes mitochondrial fitness required for metabolic adaptation of GSCs. **(A)** Heatmap of differential metabolites in control or CYP3A5-KO U87MG GSCs via non-targeted metabolomic analysis (*n* = 6). **(B)** Bar plots depicting relative abundances of TCA-related metabolites detected by non-targeted metabolomic analysis (*n* = 6). *p*-values obtained by t-test. **(C)** Gene set enrichment analysis (GSEA) of mitochondria-related pathways from the MitoCarta database using mRNA-seq data in control or CYP3A5-KO U87MG GSCs. **(D)** Seahorse XF Cell Mito Stress assay showing the altered oxygen consumption rate (OCR) in CYP3A5 KO-GSCs (*n* = 3). *p*-values obtained by one-way ANOVA. **(E)** Representative histograms and quantitative analysis of MDR staining in control and CYP3A5-KO GSCs (*n* = 3). *p*-values obtained by one-way ANOVA. **(F)** Representative electron microscope images and quantitative plots of mitochondrion number and crista density. *p*-values obtained by one-way ANOVA. Red arrows denote mitochondrion. Scale bars: 5 μm and 1 μm. **(G)** Top: Immunoblots of MT-CO2 protein in control and CYP3A5-KO GSCs. Down: The copy number of mtDNA (MT-CO2) determined by qPCR and normalized to nDNA (*n* = 3). *p*-values obtained by one-way ANOVA. **(H)** Immunoblots of acetyl-lysine levels checked on PGC1α immunoprecipitate (IP) in control and CYP3A5-KO GSCs treated with vehicle or resveratrol (RSV). The top numbers were derived by dividing the Ac-lys (IP) by PGC1α (IP). The down numbers were derived by dividing PGC1α (input) by GAPDH (input). **(I)** Immunoblots of TFAM protein in control and CYP3A5-KO GSCs treated with vehicle or RSV. **(J)** Representative histograms and quantitative analyses of MDR staining in control and CYP3A5-KO GSCs treated with vehicle or RSV (*n* = 3). *p*-values obtained by t-test. **(K)** The proportion of mitochondrial ATP in GSCs and their matched DGCs (*n* = 3); *p*-values obtained by t-test. Error bars represent mean ± SD. Significant results are presented as, ns *p* > 0.05, *p < 0.05, **p < 0.01, ***p < 0.001, ****p < 0.0001

Since NAD^+^/NADH has emerged as a crucial coenzyme capable of rewiring metabolism and maintaining mitochondrial fitness, we hypothesized that changes in the CYP3A5-mediated NAD^+^/NADH ratio alteration might regulate mitochondrial function [15]. We first conducted RNA sequencing and nontargeted metabolomic analysis to examine global transcriptional and metabolic changes, respectively. A distinct metabolite profile was observed in CYP3A5-KO GSCs, revealing a notable alteration in TCA cycle-related metabolites and a decreased NAD^+^/NADH ratio (Fig. 5A-B). To note, we observed a significant downregulation of α-ketoglutarate instead of citrate in the CYP3A5-KO cells, which could be attributed to the inhibitory effect of elevated NADH on isocitrate dehydrogenase (IDH) activity (Fig. 5B) [33]. Additionally, an analysis of RNA sequencing data revealed a significant downregulation of genes associated with the mitochondrial pathway in CYP3A5-KO GSCs (Fig. 5C, S6A). Next, we asked if CYP3A5 KO impaired the mitochondrial function of GSCs. As expected, the mitochondrial stress assay revealed a significant reduction in the oxygen consumption rate (OCR) and mitochondrial ATP synthesis in CYP3A5-KO GSCs (Fig. 5D, S6B). Additionally, CYP3A5 KO notably reduced the mitochondrial activity and membrane potential (ΔΨm), as measured by MitoTracker Deep Red (MDR) and JC-1 staining (Fig. 5E, S6C). We further examined the mitochondrial status using electron microscopy, which revealed significantly abnormal mitochondrial morphology, such as lost and whorled cristae, in CYP3A5-KO GSCs (Fig. 5F). Furthermore, CYP3A5-KO GSCs displayed decreased mitochondrial mass and fewer cristae per mitochondrion. (Fig. 5F). The decreased mitochondrial mass was also corroborated by mitochondrial confocal images, the copy number of mtDNA, and the level of mtDNA-encoded proteins (Fig. 5G, S6D-E). Consistently, the overexpression of CYP3A5 increased mitochondrial mass, as supported by elevated mtDNA levels in GSCs (Fig. S6F). Taken together, these results indicate the role of CYP3A5 in sustaining mitochondrial functions and fitness.

It is well-established that the deacetylation activity of SIRT1 is enhanced by the high NAD^+^/NADH ratio, which augments the activity of proliferator-activated receptor-gamma coactivator 1-alpha (PGC1α) to activate the transcription of mitochondrial transcription factor A (TFAM), thus facilitating mtDNA replication and mitochondrial proteins translation [34–37]. To uncover the mechanisms by which CYP3A5 sustains mitochondrial fitness, the influence of CYP3A5 on the activity of the NAD⁺/NADH-SIRT1-PGC1α-TFAM axis was then evaluated. As mentioned above, CYP3A5 has been shown to be able to regulate the NAD^+^/NADH ratio (Figs. 4J and 5B). We found that PGC1α acetylation levels were elevated in CYP3A5-KO GSCs, which could be partly reversed by supplementation with SIRT1 agonist resveratrol (RSV) (Fig. 5H). Moreover, decreased expression levels of TFAM were detected in CYP3A5-KO GSCs, which could be also reversed by replenishment with RSV (Fig. 5I). These results suggest an inhibited activity of the NAD⁺/NADH-SIRT1-PGC1α-TFAM axis in CYP3A5-KO GSCs, namely decreased NAD⁺/NADH ratio, decreased deacetylation activity of SIRT1, elevated acetylation levels of PGC1α and decreased expression TFAM. Furthermore, activation of this axis by supplementation with RSV restored the impaired mitochondrial fitness in CYP3A5-KO GSCs, suggesting that CYP3A5 regulated mitochondrial fitness through this axis (Fig. 5J). OXPHOS has emerged as a crucial metabolic pathway for GSCs [20, 38]. Consistently, in this study, higher mitochondrial ATP production was detected in GSCs than in their matched DGCs, indicating a dependency of GSCs on mitochondrial ATP (Fig. 5K). Taken together, CYP3A5 promotes the metabolic adaptation of GSCs by fine-tuning mitochondrial fitness through the NAD⁺/NADH-SIRT1-PGC1α-TFAM axis.

### CYP3A5 is transcriptionally upregulated by STAT3 in GSCs

**Fig. 6:**
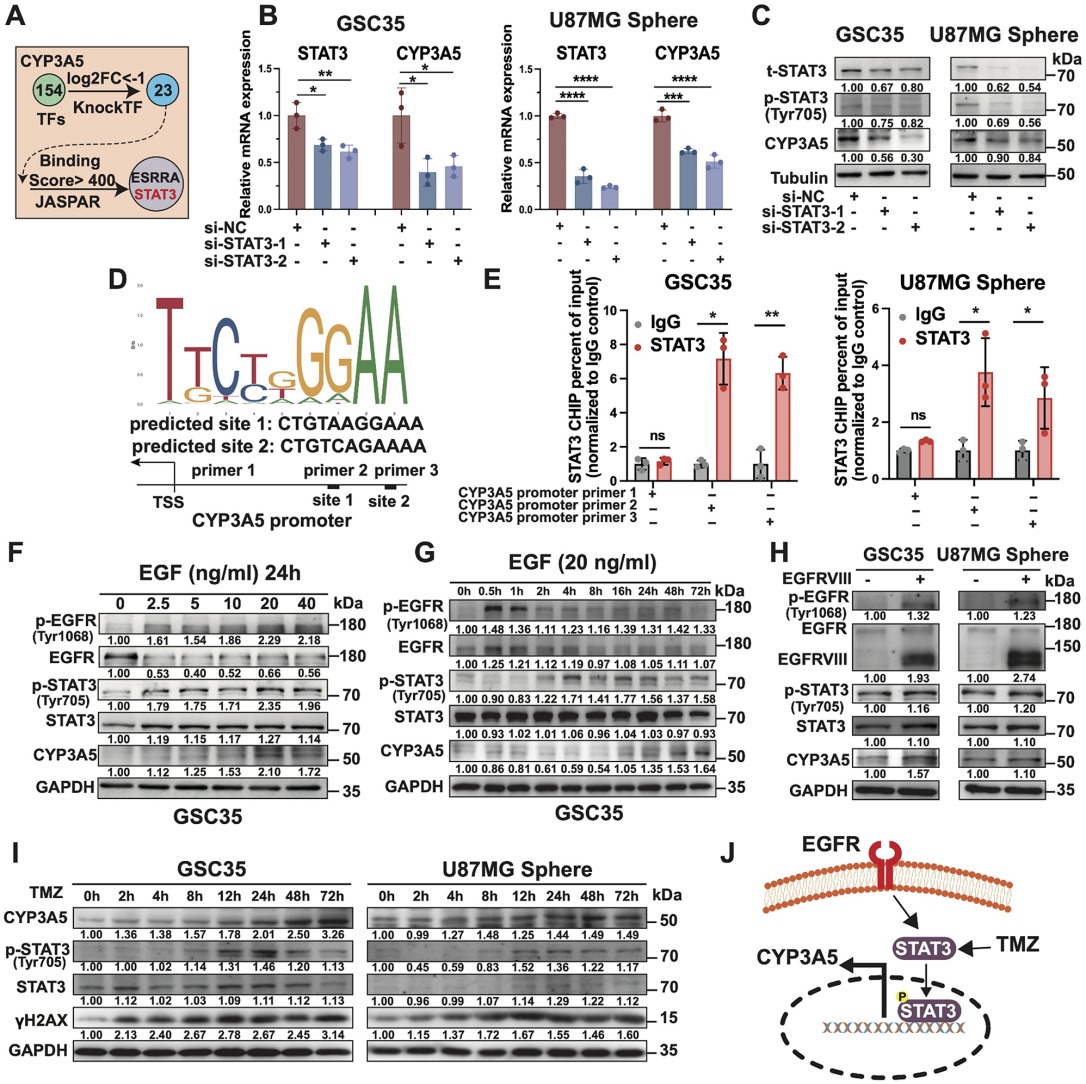
CYP3A5 is transcriptionally upregulated by STAT3 in GSCs. **(A)** The diagram depicting the strategy for the identification of potential transcription factors of CYP3A5. **(B)** qPCR analysis of STAT3 and CYP3A5 mRNA expression subsequent to knockdown with two independent siRNAs targeting STAT3 (*n* = 3). *p*-values obtained by one-way ANOVA. **(C)** Immunoblots of total STAT3, phospho-STAT3 (Try 705, p-STAT3), and CYP3A5 following knockdown with two independent siRNAs targeting STAT3. **(D)** Predicted STAT3-binding sites in the promoter region of CYP3A5 by JASPAR dataset. **(E)** Enrichment of STAT3 binding over input by ChIP-qPCR using two primers against the CYP3A5 promoter region and one negative control primer as demonstrated in D (*n* = 3). *p*-values obtained by t-test. **(F)** Immunoblots in GSCs treated with indicated concentrations of epidermal growth factor (EGF) for 24 h. **(G)** Immunoblots in GSCs treated with EGF (20 ng/ml) for varying durations. **(H)** Immunoblots in GSCs with or without EGFRVIII overexpression. **(I)** Immunoblots in GSCs treated with TMZ for varying durations. **(J)** Illustration of the postulated signaling cascade. Error bars represent mean ± SD. Significant results are presented as, ns *p* > 0.05, *p < 0.05, **p < 0.01, ***p < 0.001, ****p < 0.0001

To probe the potential transcription factors that promote CYP3A5 expression in GSCs, we interrogated the KockTF dataset and identified 23 potential transcription factors (TFs) for CYP3A5 (Fig. 6A). We analyzed these TFs using the University of California Santa Cruz (UCSC) Genome Browser and identified 2 TFs (ESRRA and STAT3) with JASPAR binding scores exceeding 400 (Fig. 6A). A higher JASPAR binding scores suggests a stronger likelihood that the TF will bind to that specific site. Since STAT3 has been reported as a TF associated with glioma stemness, we next tested whether STAT3 promoted CYP3A5 transcription [6]. Notably, the knockdown of STAT3 with two independent nonoverlapping siRNAs resulted in notable reductions in both the mRNA and protein levels of CYP3A5 (Fig. 6B-C). Blockade of STAT3 activity with the small-molecule inhibitor WP1066 also suppressed the expression of CYP3A5 (Fig. S7A). A subsequent ChIP‒qPCR analysis revealed that STAT3 exhibited increased binding affinity at the promoter region of CYP3A5 relative to a negative control site in GSCs (Fig. 6D-E, S7B). However, no obvious positive correlation between the CYP3A5 and STAT3 mRNA levels was detected in the TCGA GBM cohort, possibly due to the transcriptional activity of STAT3 is dependent on the phosphorylation of STAT3 (p-STAT3) (Fig. S7C). Collectively, these results suggest that the upregulation of CYP3A5 in GSCs is driven by STAT3 activation.

Given that EGFR signaling and TMZ have been reported to increase the level of p-STAT3, we asked whether CYP3A5 upregulation was the reaction of GSCs stimulated with EGFR signaling or TMZ treatment [5, 39]. Following overnight starvation of GSCs, epidermal growth factor (EGF) treatment notably enhanced the levels of p-STAT3 and the expression of CYP3A5 in a dose- and time-dependent manner, accompanied by activated EGFR signaling (Fig. 6F-G, S7D). EGFR signaling can be activated in a ligand-independent manner by EGFR variant III (EGFRvIII) which is present in most GBM patients. As expected, EGFRvIII overexpression in GSCs replicated the effects observed with EGF treatment (Fig. 6H). Moreover, a time course of TMZ treatment also appreciably increased the levels of p-STAT3 and CYP3A5 (Fig. 6I). Together with the aforementioned outcomes, these results suggest that when stimulated with EGFR signaling or TMZ exposure, GSCs will activate STAT3 to upregulate CYP3A5 expression (Fig. 6J).

### Pharmacological targeting of CYP3A5 impairs GBM growth and sensitizes GBM to TMZ

**Fig. 7:**
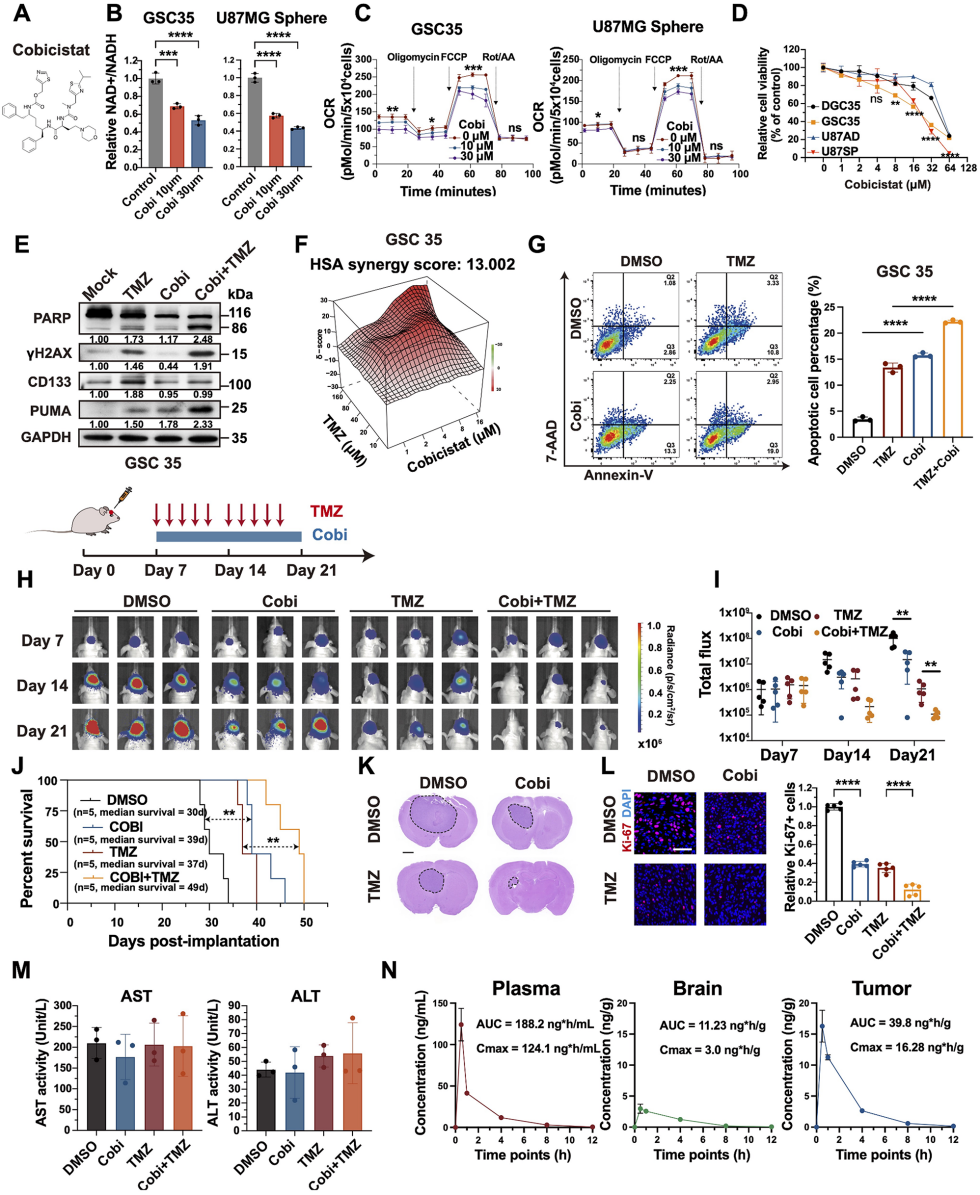
Pharmacological inhibition of CYP3A5 suppresses GBM growth and sensitizes GBM to TMZ. **(A)** Structure of Cobicistat (Cobi). **(B)** NAD-to-NADH ratios of GSCs treated with indicated concentrations of Cobi (*n* = 3). *p*-values obtained by one-way ANOVA. **(C)** Oxygen consumption rate (OCR) alterations of GSCs treated with indicated concentrations of Cobi (*n* = 3). *p*-values obtained by one-way ANOVA. **(D)** Cell viability assay with the increasing concentrations of Cobi (*n* = 3). *p*-values obtained by one-way ANOVA. **(E)** Immunoblots of cleaved PARP, γH2AX, CD133, and PUMA in GSCs treated with Cobi and TMZ. **(F)** Calculation and visualization of synergy scores for drug combinations of Cobi and TMZ. **(G)** Representative images and quantification of cell apoptosis measured by flow cytometry (*n* = 3). *p*-values obtained by one-way ANOVA. **(H-I)** Representative images and quantification of bioluminescence intensities of nude mice intracranially implanted with U87MG GSCs in treatment of DMSO, TMZ(5 mg/kg), Cobi(10 mg/kg) or TMZ(5 mg/kg) + Cobi(10 mg/kg) by gavage (*n* = 5). *p*-values obtained by one-way ANOVA. **(J)** Kaplan-Meier survival curves of the nude mice (*n* = 5). *p*-values obtained by Log-rank test. **(K)** Representative images of H&E staining of tumor sections. Scale bars, 2 mm. **(L)** Representative images and quantification of immunofluorescence staining of Ki-67 (*n* = 5). *p*-values obtained by one-way ANOVA. Scale bars, 50 μm. **(M)** AST and ALT activity in serum of mice with indicated treatment. **(N)** Liquid chromatography-tandem mass spectrometry (LC-MS) analysis of Cobi (10 mg/kg, by gavage) concentration in blood plasma, brain, and xenografts from tumor-bearing mice at indicated time points (*n* = 3 mice per group). AUC, the area under the curve. Cmax, maximum concentration. Error bars represent mean ± SD. Significant results are presented as, ns *p* > 0.05, *p < 0.05, **p < 0.01, ***p < 0.001, ****p < 0.0001

Based on the above results, we hypothesized that targeting CYP3A5 may confer therapeutic benefits against GBM progression. We thus examined the efficacy of an FDA-approved CYP3A5 inhibitor Cobicistat (Cobi) on GBM xenograft growth in mice (Fig. 7A) [40]. First, we asked if Cobi replicated the effects of CYP3A5 deficiency on NAD^+^/NADH ratio, mitochondrial fitness, stemness maintenance, and DNA damage repair in GSCs. As expected, the NAD^+^/NADH ratio was significantly decreased by Cobi in a dose-dependent manner in GSCs (Fig. 7B). Moreover, a decrease in the Oxygen consumption rate (OCR) was observed in GSCs following treatment with Cobi in a dose-dependent manner (Fig. 7C). Additionally, Cobi exerted a preferential cytotoxic effect against GSCs compared with their matched DGCs (Fig. 7D). Next, we tested the role of Cobi in DNA damage repair in GSCs. As expected, Cobi augmented TMZ-induced cell apoptosis and DNA damage, as shown by elevated levels of cleaved PARP and γH2AX (Fig. 7E, S8A). Moreover, Cobi also augments DNA damage induced by TMZ in high MGMT-expressing cell line T98G (Fig. S8B). TMZ has been reported to be able to mediate glioma stemness [41]. Notably, Cobi reversed the upregulation of the stemness marker CD133 induced by TMZ (Fig. 7E, S8A). Given Cobi inhibits mitochondrial fitness and exerts cytotoxicity on GSCs, we wondered whether the use of Cobi would enhance GSC mitochondrial apoptosis which is mediated by mitochondrial outer membrane permeabilization protein PUMA. Indeed, Cobi increased the PUMA levels induced by TMZ (Fig. 7E, S8A). Together, these results suggest that Cobi replicates the effect of CYP3A5 deficiency on restraining GSCs and augments the efficacy of TMZ.

Next, we tested the synergistic benefits of the combinatorial use of Cobi with TMZ. Analysis of drug combination dose-response matrix data and flow cytometry apoptosis assays revealed a synergistic effect of Cobi and TMZ on inducing GSCs apoptosis(Fig. 7F-G, S8C-D). Similar results were corroborated using another CYP3A5-selective inhibitor, clobetasol propionate (CP) (Fig. S8E-I) [42]. Next, we determined the efficacy of combining Cobi and TMZ for GBM treatment in vivo. Notably, in established orthotopic GBM xenograft models, the coadministration of Cobi and TMZ exhibited superior efficacy in suppressing tumor growth and prolonging overall survival compared with individual Cobi or TMZ treatment alone (Fig. 7H-K). Immunofluorescence staining of tumor sections revealed a pronounced decrease in the expression of the proliferation marker Ki67 following the combination treatment (Fig. 7L). Furthermore, the administration of Cobi and TMZ, either as monotherapies or in combination, did not elicit any observable signs of toxicity, based on assessments of the serum levels of aspartate aminotransferase (AST) and alanine aminotransferase (ALT) and histological staining of the liver, kidney, or heart (Fig. 7M, S8J). To note, an analysis of drug distribution revealed higher concentrations of Cobi in brain tumor tissue than in nontumor brain tissue following delivery into plasma (Fig. 7N). Collectively, these results demonstrate that Cobi able to be delivered into brain tumors exerts a synergistic effect with TMZ on restraining GBM growth in a safe manner.

Taken together, these data show that CYP3A5 represents a promising therapeutic target in combination with TMZ for GBM treatment.

## Discussion

The cellular and molecular mechanisms by which GSCs respond to various cell stimuli to maintain self-renewal and develop treatment resistance remain incompletely elucidated. In this study, we identify a novel GSC target CYP3A5 based on the glioma stemness-related score system GScore. We find that EGFR signaling induced by EGF or EGFRVIII, or the cytotoxic pressure induced by TMZ, can activate STAT3 to enhance the transcription of CYP3A5 in GSCs. Further loss of function and gain of function genetic experiments consolidate the role of CYP3A5 in stemness maintenance and TMZ resistance in GSCs. Mechanistic studies reveal that CYP3A5 promotes mitochondrial fitness to facilitate GSC metabolic adaptation via the NAD^+^/NADH-SIRT1-PGC1α-TFAM axis. Additionally, CYP3A5 promotes the activity of NAD^+^-dependent PARP to enhance DNA damage repair of GSCs. These findings indicate that CYP3A5 may serve as a mediator in the development of malignant phenotypes of GSCs as they adapt to various intrinsic and extrinsic factors. We believe this insight will facilitate the development of effective inhibitors targeting GSCs for the treatment of glioblastoma.

The NAD^+^/NADH ratio is a critical factor for influencing tumor progression. Mitochondrial complex I can convert NADH to NAD^+^ to facilitate kidney cancer metastasis and this effect is replicated by ectopic expression of yeast mitochondrial NADH dehydrogenase NDI1 and NADH oxidase from Lactobacillus brevis (LbNOX) [19]. Similarly, ectopic expression of NDI1 for a higher NAD^+^/NADH ratio is sufficient to trigger brain tumor cell immortalization in a Drosophila neural stem cell (NSC)-derived brain tumor model [20]. Therefore, manipulation of the NAD^+^/NADH ratio is a promising strategy to restrain tumor growth. SLC25A51 increases the mitochondrial NAD^+^/NADH ratio, thus controlling the proliferation of acute myeloid leukemia cells [43]. ALDH3A1 reduces NAD^+^ to NADH, hence inducing metabolic liability in NRF2-activated lung cancers [44]. MDHDH promotes the degradation of mitochondrial malate dehydrogenase2 (MDH2) to reduce the NAD^+^/NADH ratio, thus suppressing GSC growth [45]. We here show that the CYP3A5 enzyme increases the NAD^+^/NADH ratio to sustain stemness maintenance and chemoresistance of GSCs. Therefore, our study identifies a new insight into manipulating the NAD^+^/NADH ratio to impact GSC malignant features.

Metabolic plasticity enables CSCs to dynamically alter their energy utilization pathways in response to various environmental and extrinsic factors. Mounting evidence indicates that OXPHOS seems indispensable for GSCs to meet their energy needs for diverse cellular processes. Using cryo-electron tomography, Wang et al. reported that more inter-mitochondrial junctions were detected in GBM compared with cancer-free brain cells [46]. The upregulation of mitochondrial OXPHOS facilitates the immortalization of brain neural stem cells during the brain tumorigenic process [20, 38]. Our data also revealed that GSCs were more dependent on mitochondrial OXPHOS to generate ATP than their matched DGCs were. These results suggest that mitochondrial targeting may be a potential strategy for eradicating GSCs, and a series of attempts, including the inhibition of respiratory complex activity and assembly to suppress GSCs, have been reported [10, 47]. Here, we find that CYP3A5 enhances GSC mitochondrial fitness and functions via the NAD^+^/NADH-SIRT1-PGC1α axis, thus contributing to GSC metabolic adaptation and growth. Additionally, treatment of CYP3A5 inhibitor augments the expression of mitochondrial apoptosis-related protein PUMA to promote GSC apoptosis. Therefore, our study identifies a new target for mitochondrial fitness intervention to suppress GSCs.

The use of TMZ has long been a standard treatment for GBM patients, yet it only provides a limited increase in survival time, mainly due to its intrinsic and acquired resistance. Gaining insight into the mechanisms of TMZ chemoresistance is essential for improving treatment results. CYP3A5 has been proposed to be a key mediator of both intrinsic and acquired chemoresistance in pancreatic ductal adenocarcinoma, possibly because of its involvement in tumor cell-autonomous drug detoxification [24, 25]. GSCs have been identified to develop enhanced DNA damage repair capabilities during the GBM treatment and contribute to therapy resistance [3]. PARP1 is essential for DNA repair, catalyzing the transfer of ADP-ribose polymers to various downstream substrates, including numerous DNA repair enzymes. This process plays a critical role in the repair of DNA damage, particularly in response to single-strand and double-strand breaks. Here, we found that GSCs preferentially elevate the expression of CYP3A5 during exposure to TMZ treatment and subsequently mitigate the DNA damage. In addition, we reported a novel way of CYP3A5 in mediating tumor chemoresistance, which is mediated by modulating NAD+/NADH levels to impact PARP1 DNA repair activity.

The CYP3A enzyme subfamily, which is part of the cytochrome P450 (CYP) superfamily, encompasses isoforms such as CYP3A4, CYP3A5, CYP3A7, and CYP3A43 [48]. CYP3A enzymes are responsible for the metabolism of endogenous compounds, such as steroid hormones, bile acids, polyunsaturated fatty acids, and xenobiotics [48]. Additionally, these enzymes have been implicated in a spectrum of pathological conditions, including tumorigenesis. For example, CYP3A4 has been reported to increase the growth of breast cancer by facilitating the nuclear translocation of p-STAT3 partially through the synthesis of (±)-14,15-epoxyeicosatrienoic acid (EET) [49]. Moreover, the epoxygenase activity of CYP3A4 on arachidonic acid (AA), which is required for breast tumor formation, promotes the activity of the mitochondrial electron transport chain, which can be inhibited by metformin [50]. However, the role of CYP3A5 in cancer progression is context-dependent. CYP3A5, which is downregulated in hepatocellular carcinomas, has been found to suppress HCC pathogenesis by inducing ROS accumulation and subsequent inhibition of mTORC2/Akt signaling [51]. Moreover, lung cancer metastasis is inhibited by CYP3A5 through regulation of the ATOH8/Smad1 axis [52]. Conversely, the inhibition of CYP3A5 has been shown to suppress prostate cancer growth by increasing the nuclear translocation of the androgen receptor [53]. Our data suggest that the expression of CYP3A5 is significantly elevated in GSCs and is associated with a poor prognosis for GBM patients receiving TMZ treatment or not. We also reveal that CYP3A5 deficiency impairs stemness maintenance and TMZ resistance by modulating the NAD^+^/NADH-SIRT1-PGC1α axis and the activity of PARP, respectively. These data, along with our findings, provide a rationale for the research on uncovering the roles of CYP3A in tumor progression and therapy resistance.

GBM is the most lethal primary brain cancer that inevitably recurs. Recently, the emergence of immunotherapy and TTFields has improved the prognosis of GBM patients [2, 54]. However, the clinical outcome of GBM patients remains dismal, which is partly attributable to the therapeutic resistance of GSCs; thus, new treatments targeting GSCs are needed. Here, we report a novel target, CYP3A5, which contributes to the self-renewal and chemoresistance of GSCs. Due to its nonessential role in normal physiological functions, targeting CYP3A5 represents a promising therapeutic avenue [55]. In this study, we determine that Cobi, a potent CYP3A5 inhibitor, potently impedes DNA repair and mitochondrial OXPHOS, and exerts a preferential toxic effect on GSCs. The combined use of Cobi and TMZ notably augmented TMZ-induced cell apoptosis and DNA damage. It is noteworthy that Cobi effectively penetrates into brain tumors, and in combination with temozolomide (TMZ), it inhibits the growth of orthotopic GBM xenografts without any observable side effects. Therefore, this combinatorial regimen provides a promising regime for GBM treatment.

**Fig. 8:**
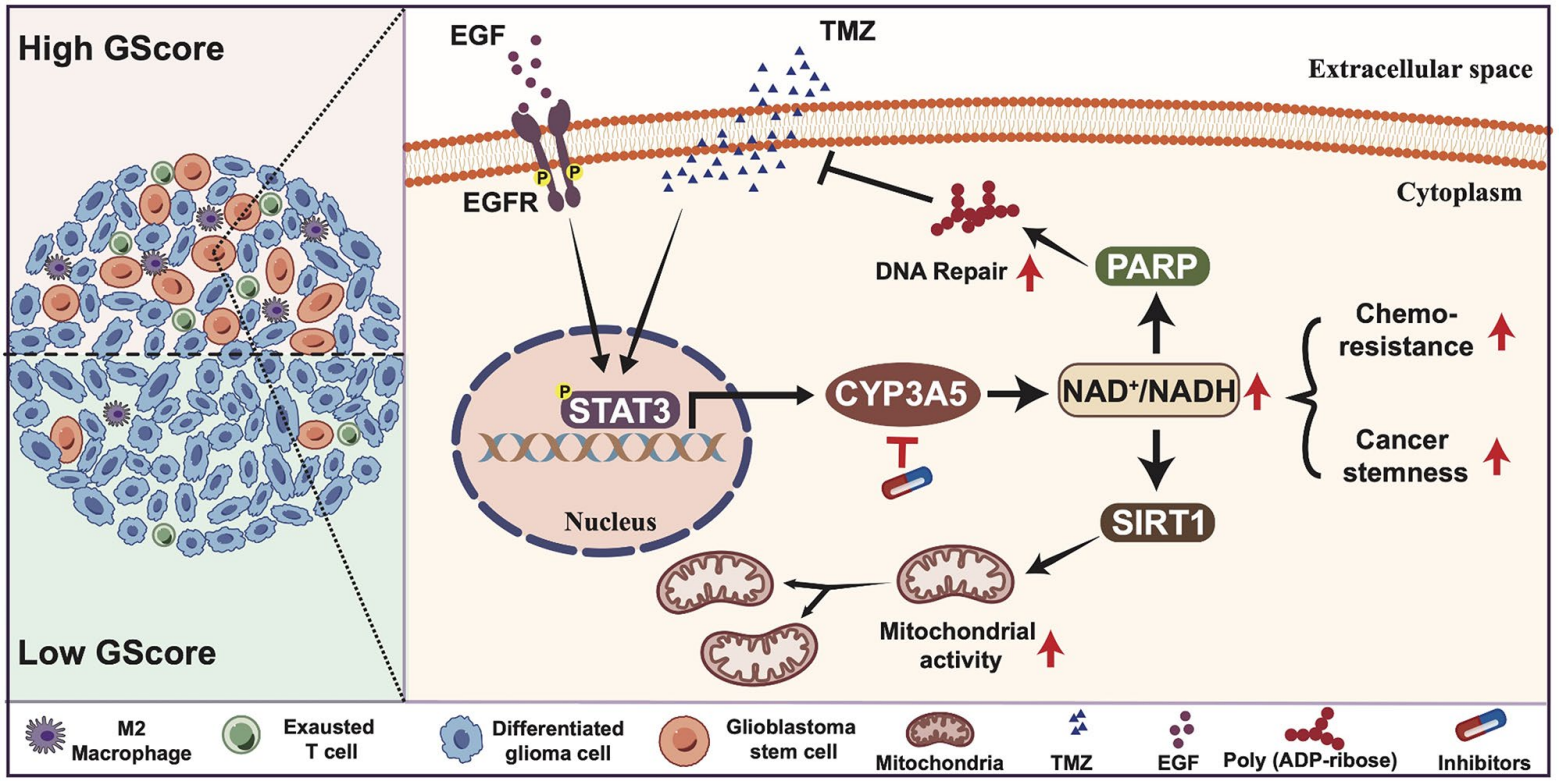
The CYP3A5 target in glioblastoma stem cells. The mechanistic scheme elucidates the pivotal role of CYP3A5 in fostering glioblastoma stemness and chemoresistance by regulating the NAD⁺/NADH balance. EGFR signaling and TMZ treatment activate STAT3, which in turn upregulates the transcriptional expression of CYP3A5 in glioblastoma stem cells (GSCs). The elevated CYP3A5 subsequently increases the NAD⁺/NADH ratio, thereby augmenting the activity of poly(ADP-ribose) polymerase (PARP) and sirtuin 1 (SIRT1) to facilitate DNA repair and optimize mitochondrial fitness, respectively. Collectively, these findings underscore that GSCs upregulate CYP3A5 to fine-tune the NAD^+^/NADH ratio for survival advantages and highlight targeting CYP3A5 as a potential strategy for GBM treatment

## Conclusions

In conclusion, our research provides valuable insights into the metabolic adaptation that GSCs exploit to enhance stemness maintenance and chemoresistance in response to various cellular stimuli. We identify the pivotal role of CYP3A5 in this process and thus establish a robust foundation for future investigations aimed at leveraging the therapeutic potential of CYP3A5 in GBM treatment (Fig. 8).

## Electronic supplementary material

Below is the link to the electronic supplementary material.

Supplementary Material 1

Supplementary Material 2

Supplementary Material 3

## Data Availability

All relevant raw data will be freely available to any researcher for non-commercial purposes on request. RNA-seq of this study data is available in Gene Expression Omnibus (GEO) with accession code GSE277539. All untargeted metabolomic data used in this publication have been deposited to the EMBL-EBI MetaboLights database with the identifier MTBLS11100.

## Abbreviations

GBM: Glioblastoma multiforme
GSC: Glioblastoma stem cell
DGC: Differentiated glioblastoma cells
TMZ: Temozolomide
MGMT: O6-methylguanine methyltransferase
OS: Overall survival
OXPHOS: Oxidative phosphorylation
CSC: Cancer stem cell
NAD: Nicotinamide adenine dinucleotide
NADP: Nicotinamide adenine dinucleotide phosphate
SIRTs: Sirtuins
PAR: Poly(ADP-ribose)
PARP: Poly(ADP-ribose) polymerase
CYP: Cytochrome P450 enzyme
GScore: Glioma stemness-related score
IDH: Isocitrate dehydrogenase
GSEA: Gene Set Enrichment Analysis
IHC: Immunohistochemical
TF: Transcription factor
EGFR: Epidermal Growth Factor Receptor
EGF: Epidermal Growth Factor
UCSC: University of California Santa Cruz
TCGA: The Cancer Genome Atlas
CGGA: Chinese Glioma Genome Atlas
ChIP: Chromatin immunoprecipitation
OCR: Oxygen consumption rate
ssGSEA: Single sample Gene Set Enrichment Analysis
MDR: MitoTracker Deep Red
WGCNA: Weighted gene co-expression network analysis
LASSO: The least absolute shrinkage and selection operator
NMN: Nicotinamide mononucleotide
mtDNA: Mitochondrial DNA
RSV: Resveratrol
Cobi: Cobicistat
AST: Aspartate aminotransferase
ALT: Alanine aminotransferase
