# Supplementary material for: CYP3A5 promotes glioblastoma stemness and chemoresistance through fine-tuning NAD+/NADH ratio
Source: J Exp Clin Cancer Res. 2025 Jan 3;44:3. doi: 10.1186/s13046-024-03254-x (PMC11697892; doi:10.1186/s13046-024-03254-x)
Supplement: Supplementary file 3 — Supplementary Material 3 [file 13046_2024_3254_MOESM3_ESM.docx]

**Supplementary Figure S1**

**
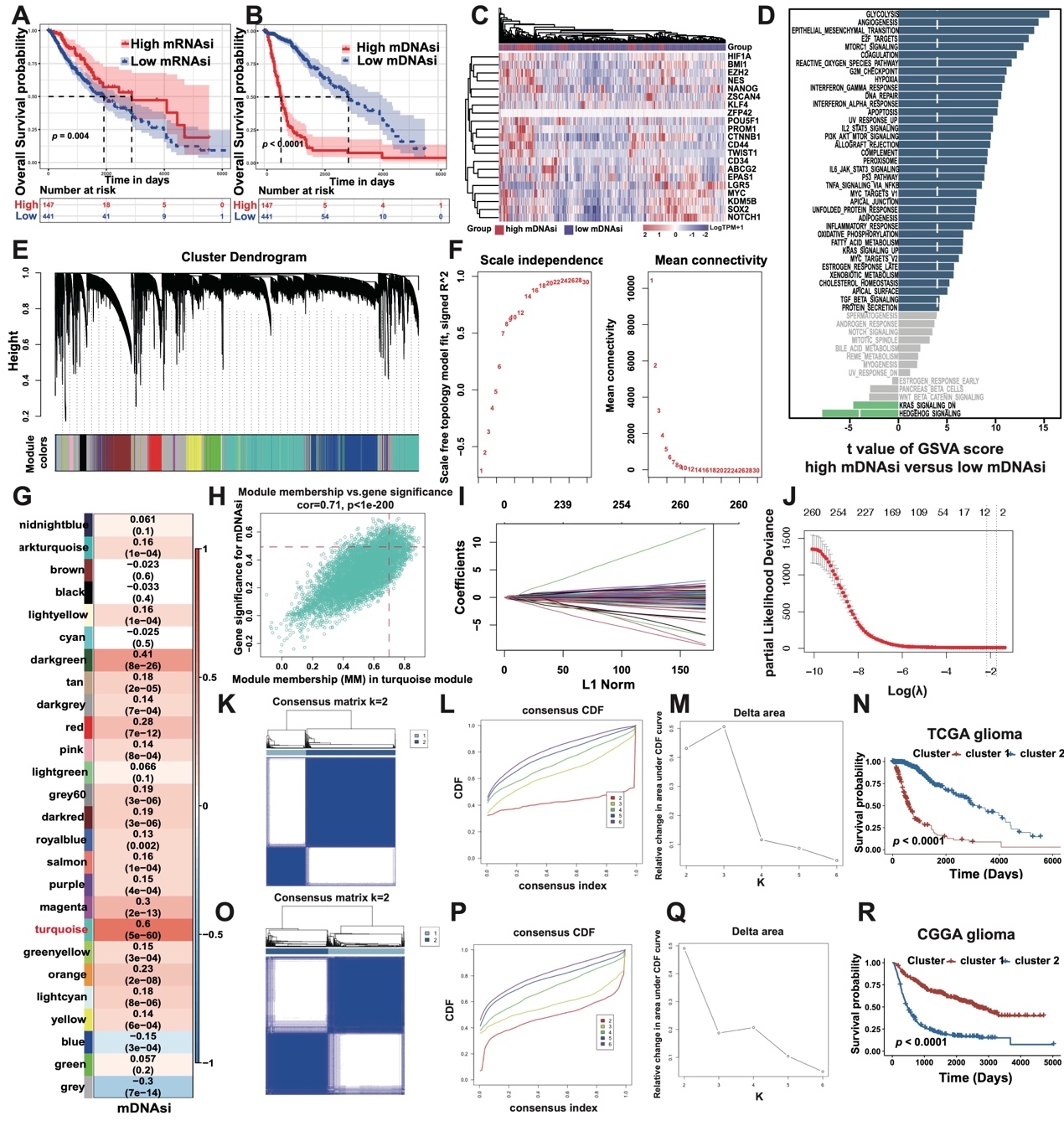
**

**Supplementary Figure S1. Construction of glioma stemness-related score (GScore)**

**(A-B)** Kaplan–Meier analysis of RNA-seq data of TCGA glioma samples sorted by the quartile of stemness index. *p* values were obtained by log-rank test. **(C)** Heatmap with unsupervised clustering analysis of the TCGA glioma cohort grouped by mDNAsi based on stemness-related genes listed vertically. **(D)** Differential analysis of pathways activities obtained from Hallmark gene sets of MsigDB. Pathway activities were scored per sample by GSVA between high mDNAsi and low mDNAsi groups. Shown are t values from a linear model. **(E)** Clustering dendrograms of different gene modules were shown in different colors based on the dissimilarity measurement from WGCNA analysis. **(F)** Analysis of the scale-free index for various soft-thresholding powers (β). Numbers in the plots denoted the corresponding β. The soft-thresholding power of 14 was determined to obtain the approximate scale-free network topology. **(G-H)** A total of 26 modules were identified by WGCNA. The heatmap showed that the turquoise module was most significantly correlated with mDNAsi with a correlation of 0.60 and a *p*-value of 5e-60; The scatterplot showed gene significance for mDNAsi in the turquoise module. **(I)** This plot displayed the magnitude of the coefficients against the regularization parameter (L1 Norm) from the LASSO model. **(J)** The plot illustrated the cross-validation process for picking the lambda value in lasso regression. **(K-R)** The consensus clustering matrix was shown with an optimal cluster number of two for TCGA glioma samples (K) or CGGA glioma cohorts (O). Consensus clustering diagnostic plots of cumulative distribution function (CDF) curves TCGA glioma samples (L-M) or CGGA glioma cohorts(P-Q). Kaplan–Meier analysis showed a distinct prognosis of patients in Cluster 1 and Cluster 2 (N, R). *P* values were obtained by log-rank test.

**Supplementary Figure S2**


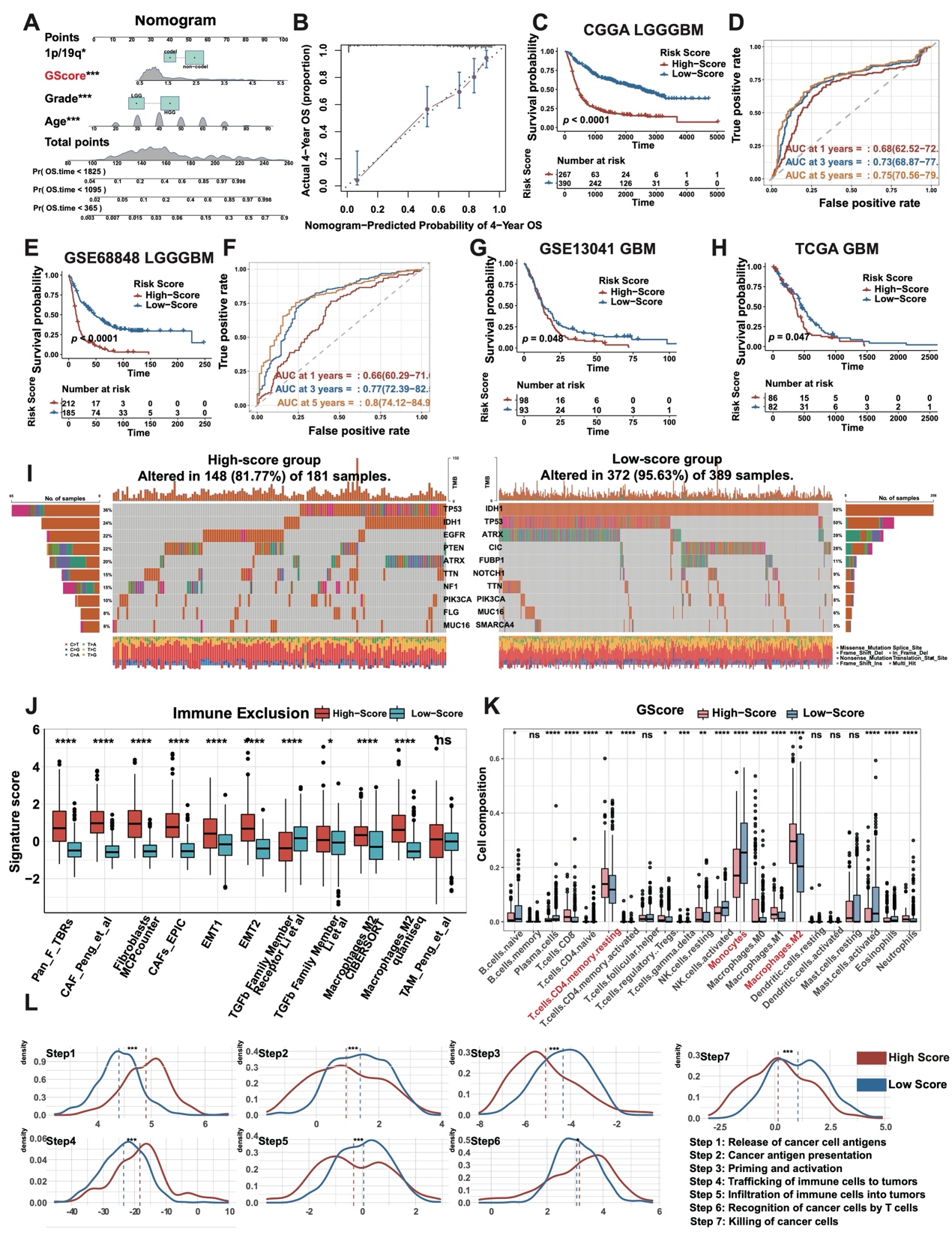


**Supplementary Figure S2. GScore associated with glioma progression and immune landscape**

**(A-B)** Details of the nomogram and accuracy of the nomogram by calibration analysis. **(C-H)** The Kaplan–Meier and time-dependent ROC curves of glioma samples stratified by GScore. The *P*-values were obtained by the Log-rank test. **(I)** Oncoplots showed the top 10 mutated genes in each GScore group. The right and left showed mutation percentages, and the top showed the overall mutation burden of each patient. The color coding denoted the mutation type. **(J)** The boxplots showed the differences in signature scores of several immune exclusion-related gene sets collected by the IOBR database by the ssgsea method. The *P*-values were obtained by the Mann-Whitney U test. **(K)** Comparison of 22 immune-infiltrating cell abundances between two groups stratified by GScore based on the CIBERSORTx algorithm in the TCGA cohort. The *P*-values were obtained by the Mann-Whitney U test. **(L)** The density plots delineated differences in immune activity scores of the seven-step cancer-immunity cycle. The *P*-values were obtained by the Mann-Whitney U test. Significant results are presented as, ns *P*>0.05, **P* < 0.05, ***P* < 0.01, ****P* < 0.001, *****P* < 0.0001.

**Supplementary Figure S3**


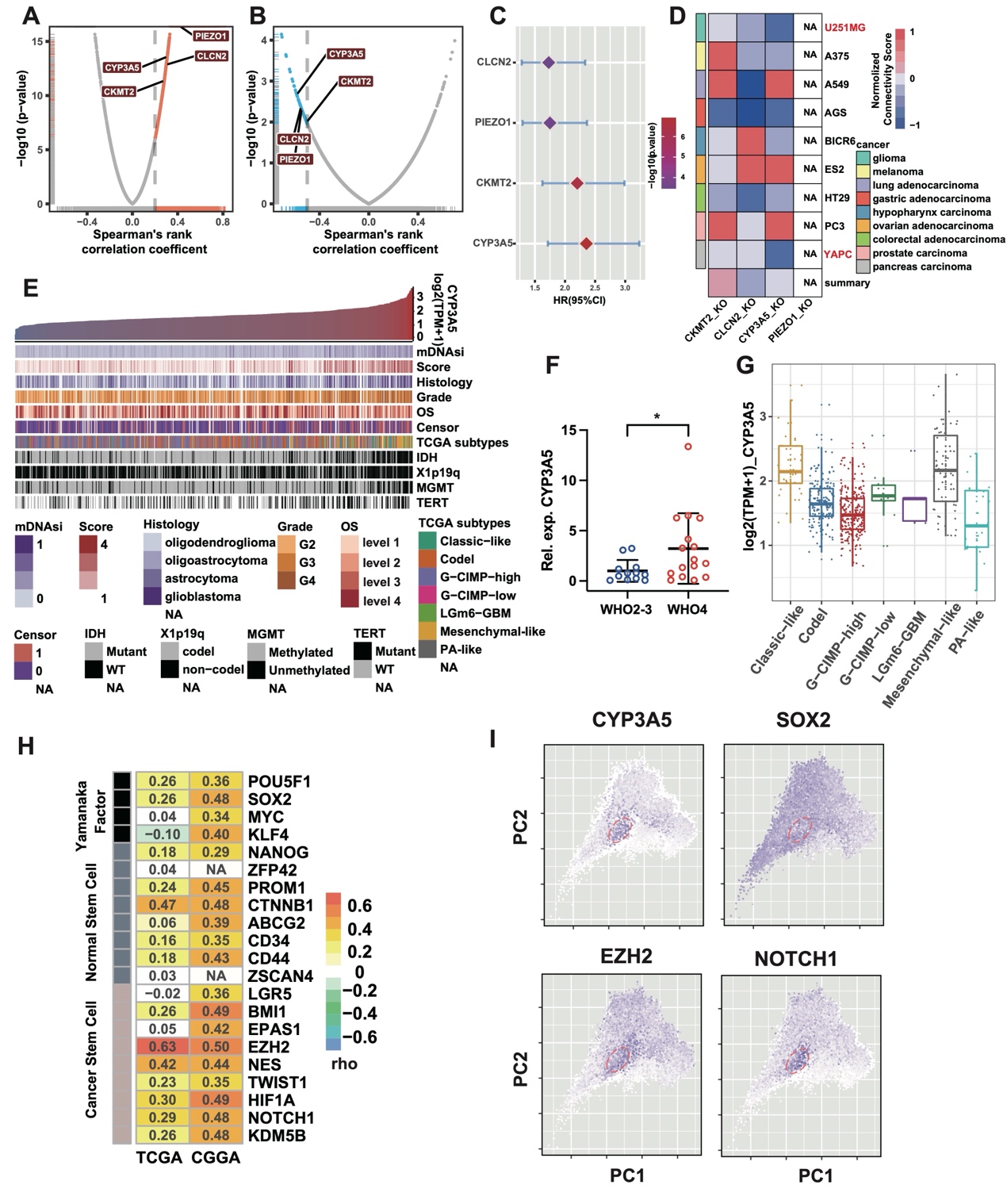


**Supplementary Figure S3. Screened CYP3A5 target associated with GBM progression and stemness**

**(A)** The volcano plot delineated the Spearman correlation between GScore and gene expression levels of four candidate targets in the TCGA GBMLGG dataset. Red dots denoted the significant positive correlations (*P* < 0.05 and Spearman’s rho > 0.2). **(B)** The volcano plot exhibited the Spearman correlation between GScore and Chronos scores of four candidate targets in Achilles glioma cell lines from CCLE. Blue dots denoted the significant negative correlations (*P* < 0.05 and Spearman’s r < −0.5). **(C)** The forest plot of single covariate Cox proportional hazards analysis of four candidate targets in TCGA GBMLGG samples. **(D)** The heatmap showed the connectivity scores of four candidate targets to high GScore for 9 CCLE-derived cancer cell lines. **(E)** The overview of CYP3A5 expression among several clinical and molecular characteristics in the RNA-seq data of TCGA GBMLGG cohort. Columns represent samples sorted by CYP3A5 expression levels from low to high. Rows represent molecular and biological processes associated with CYP3A5. **(F)** Evaluation of CYP3A5 mRNA levels via RT-qPCR among WHO 4 (n = 16) and WHO 2-3 (n = 12) gliomas in the PLAGH-glioma cohort. Data were represented as mean ± SEM. *P* values were obtained by the Mann-Whitney test. **(G)** The expression of CYP3A5 among different glioma subtypes. **(H)** Heatmap of Spearman rank correlation coefficient of CYP3A5 with stemness-related gene expression in TCGA and CGGA glioma cohorts. **(I)** PCA plots of single-cell RNA-sequencing data of 65,655 GSCs and 14,207 GBM cells. Each dot denoted a cell sample. Color represents the mRNA expression level. Error bars represent mean ± SD. Significant results are presented as, ns *P*>0.05, **P* < 0.05, ***P* < 0.01, ****P* < 0.001, *****P* < 0.0001.

**Supplementary Figure S4**


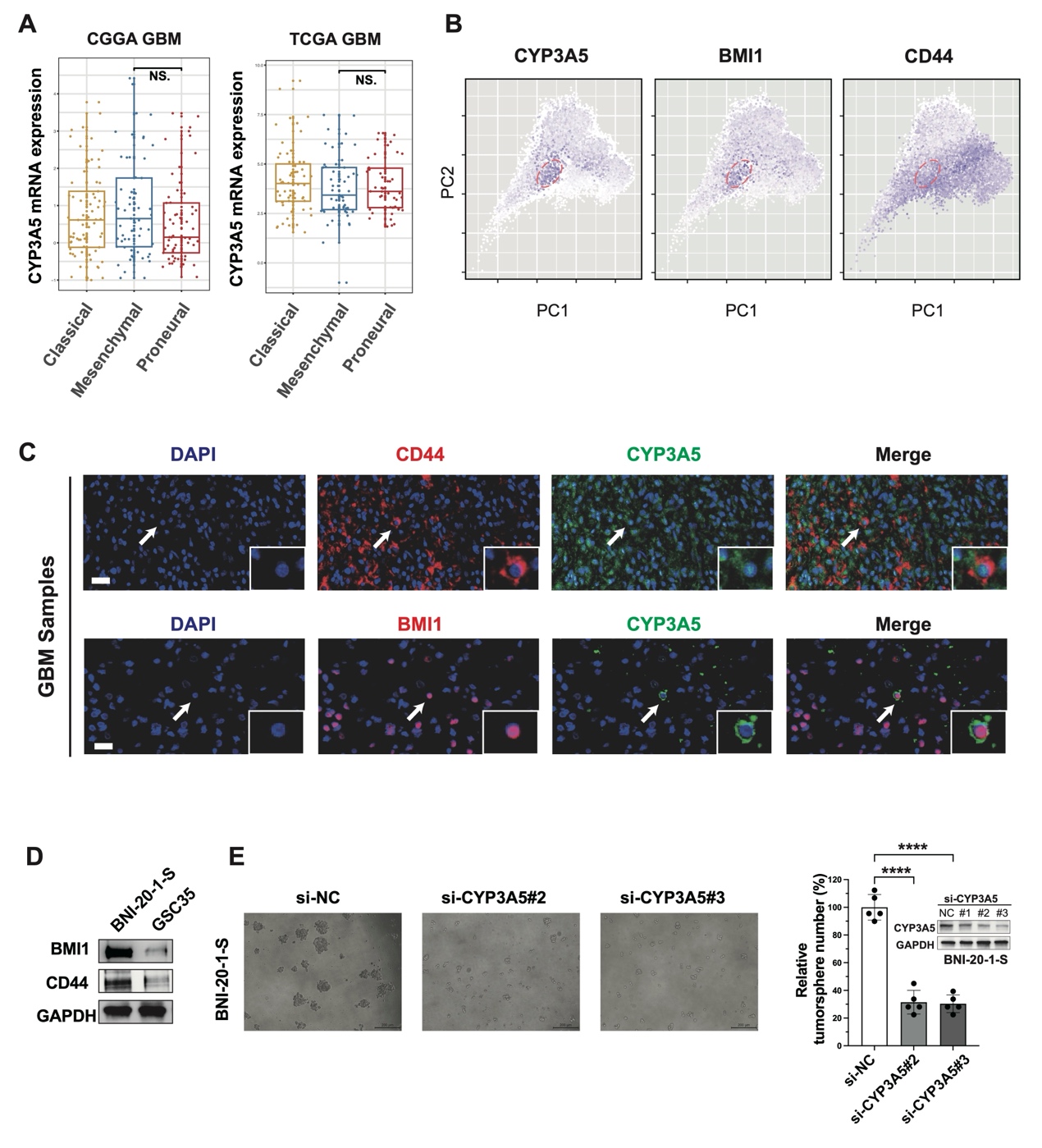


**Supplementary Figure S4. CYP3A5 is expressed by mesenchymal GSCs.**

**(A)** The boxplots show the expression of CYP3A5 across different GBM subtypes. p-values obtained by the Mann-Whitney U test. **(B)** PCA plots of single-cell RNA-sequencing data of 65,655 GSCs and 14,207 GBM cells. Each dot denoted a cell sample. Color represents the mRNA expression level. **(C)** Co**-**immunofluorescence analysis of CYP3A5 with relation to CD44 and BMI1 in GBM samples. Scale bar: 20 μm. **(D)** Immunoblot analysis of BMI1 and CD44 in different GSC lines. **(E)** Neurosphere formation assay of BNI-20-1-S after CYP3A5 knowdown (n = 5). p-values obtained by the one-way ANOVA test. Error bars represent mean ± SD. Significant results are presented as, ns *P*>0.05, **P* < 0.05, ***P* < 0.01, ****P* < 0.001, *****P* < 0.0001.

**Supplementary Figure S5**


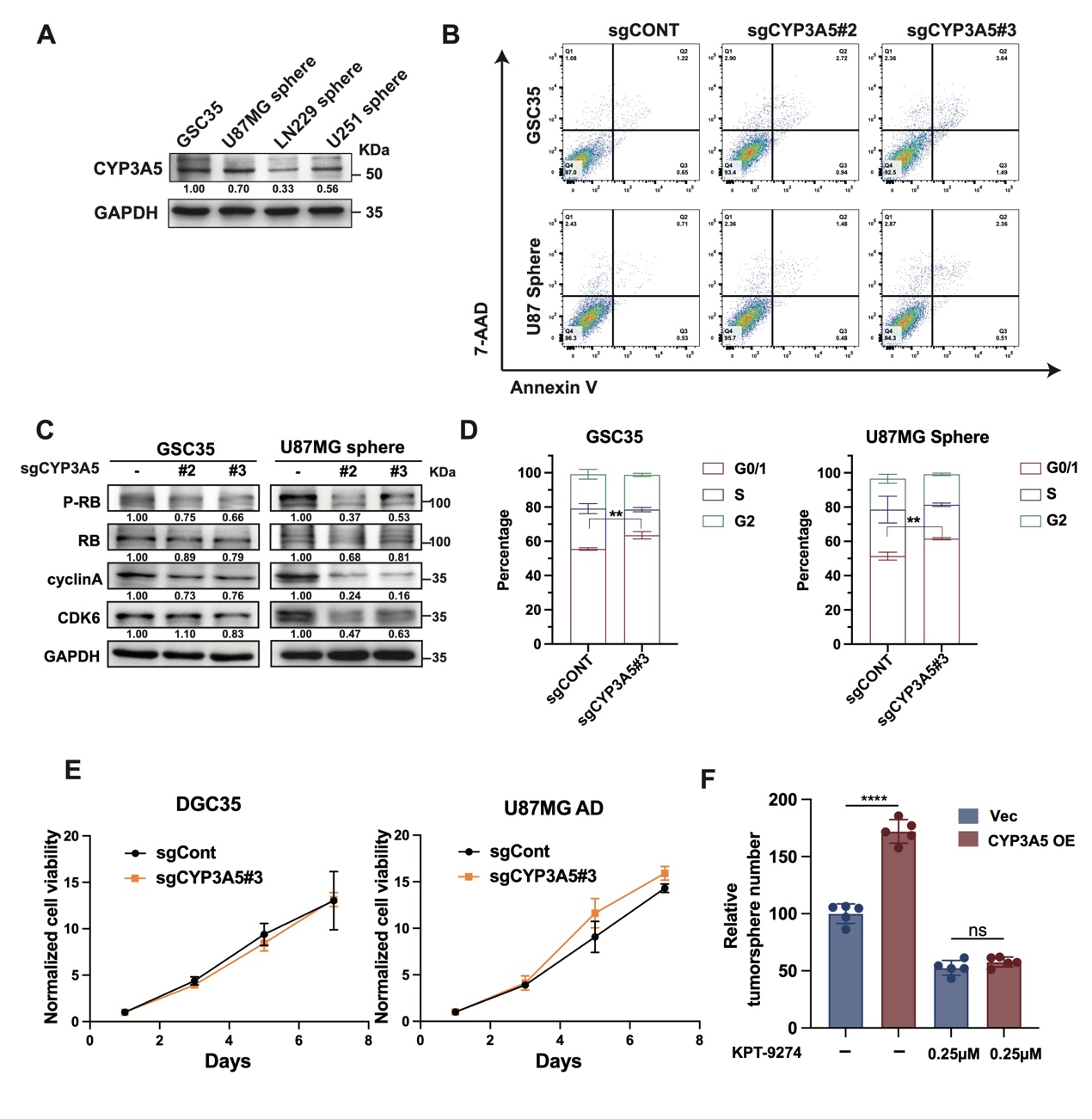


**Supplementary Figure S5. CYP3A5 sustains self-renewal of GSCs.**

**(A)** Immunoblot of CYP3A5 across various cell lines. **(B)** Cell apoptosis by Flow cytometry in GSCs with or without CYP3A5 KO. **(C-D)** Immunoblot of cell cycle-related proteins and cell cycle analysis of Flow cytometry in GSCs with or without CYP3A5 KO. The *P*-values were obtained by t-test. **(E)** Cell viability assay of DGCs with or without CYP3A5 KO. (**F**) Relative tumorsphere number in CYP3A5 overexpressing GSCs treated with or without KPT-9247. The *P*-values were obtained by t-test. Error bars represent mean ± SD. Significant results are presented as, ns *P*>0.05, **P* < 0.05, ***P* < 0.01, ****P* < 0.001, *****P* < 0.0001.

**Supplementary Figure S6**

**
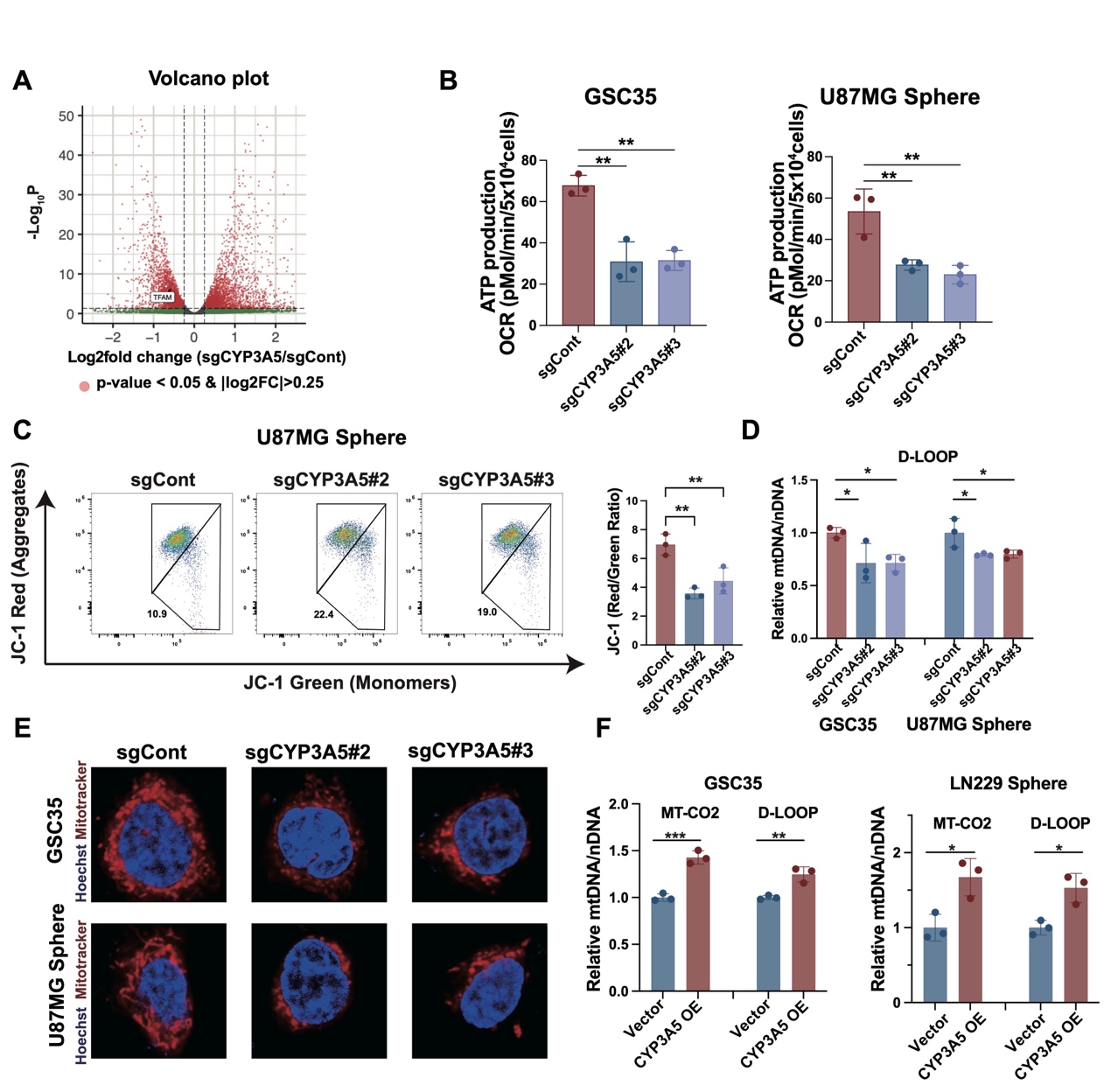
**

**Supplementary Figure S6. CYP3A5 fine-tunes mitochondrial fitness required for metabolic adaptation of GSCs**

**(A)** The volcano plot depicted the differential expressed genes (DEGs) between CYP3A5 KO and control U87MG GSCs.(**B**) Seahorse XF Cell Mito Stress assay showing the altered ATP production in CYP3A5 KO-GSCs (n = 3). *p*-values obtained by one-way ANOVA. **(C)** Representative images and quantification of JC-1 staining detected by Flow cytometry in GSCs with or without CYP3A5 KO. *P* values were obtained by one-way ANOVA. **(D)** The alteration in mtDNA content by measuring D-LOOP copy number by qPCR normalized to nuclear DNA amount. *P* values were obtained by one-way ANOVA **(E)** Representative immunofluorescence images of MitoTracker Deep Red (MDR) immunostaining. Scale bar: 5µm. **(F)** The alteration in mtDNA content by measuring MT-CO2 or D-LOOP copy number by qPCR normalized to nuclear DNA amount when overexpressing CYP3A5. *P* values were obtained by t-test. Error bars represent mean ± SD. Significant results are presented as, ns *P*>0.05, **P* < 0.05, ***P* < 0.01, ****P* < 0.001, *****P* < 0.0001

**Supplementary Figure S7**


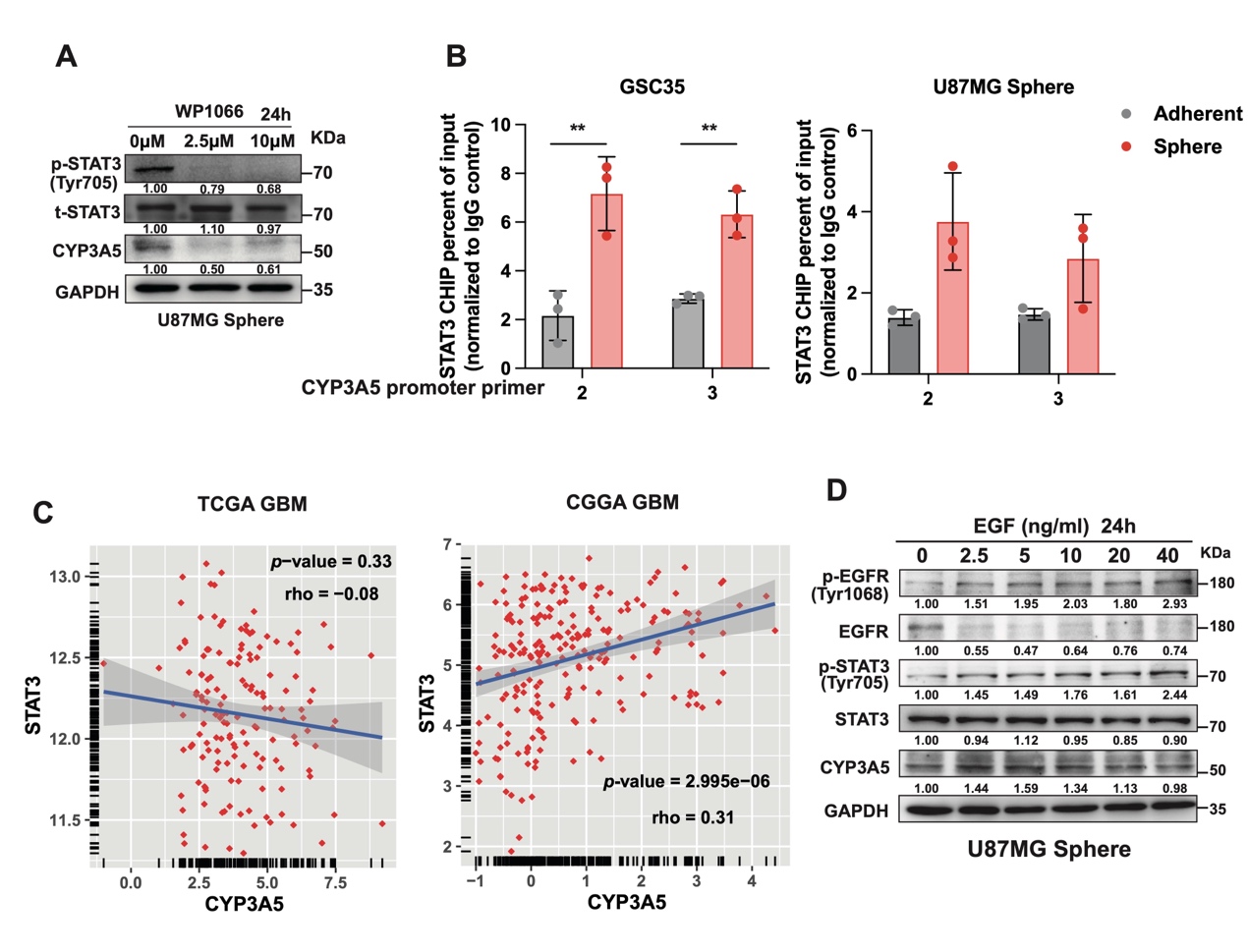


**Supplementary Figure S7. CYP3A5 is transcriptionally upregulated by STAT3 in GSCs**

**(A)** Immunoblot of total STAT3, p-STAT3, and CYP3A5 in GSCs treated with indicated concentrations of WP1066 for 24h, an inhibitor of STAT3 activity. **(B)** Enrichment of STAT3 binding over input by ChIP-qPCR using two primers against the CYP3A5 promoter region in GSCs and their matched DGCs. *P* values were obtained by t-test. **(C)** Spearman correlation among CYP3A5 and STAT3 in TCGA GBM and CGGA GBM cohorts. *P* values were obtained by the Spearman correlation test. **(D)** Immunoblot in U87MG spheres treated with indicated concentrations of epidermal growth factor (EGF). Error bars represent mean ± SD. Significant results are presented as, ns *P*>0.05, **P* < 0.05, ***P* < 0.01, ****P* < 0.001, *****P* < 0.0001.

**Supplementary Figure S8**


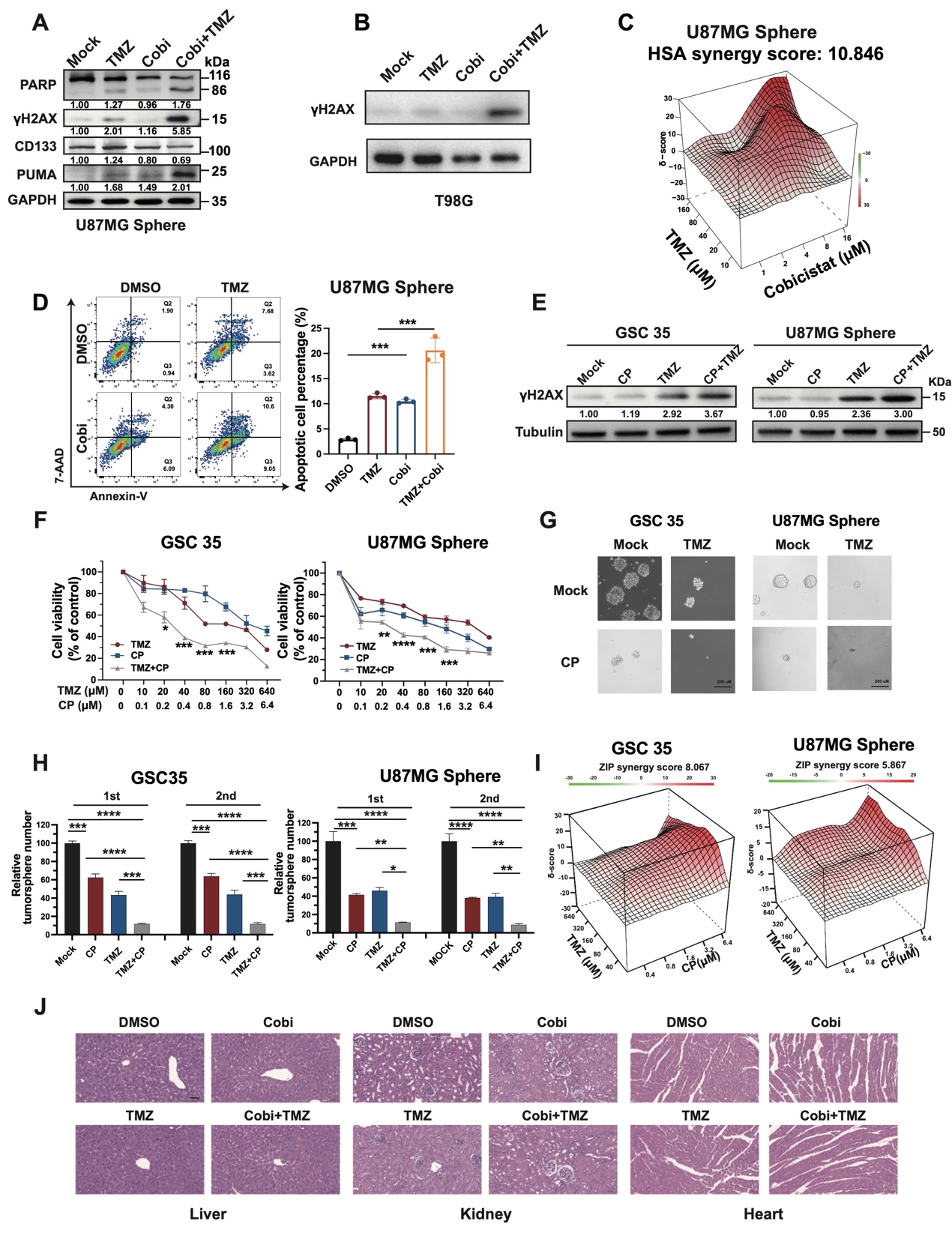


**Supplementary Figure S8. Pharmacological targeting of CYP3A5 impairs mitochondrial functions and sensitizes tumors to TMZ**

**(A)** Immunoblots of cleaved PARP, γH2AX, CD133, and PUMA in GSCs treated with Cobi and TMZ. **(B)** Immunoblot of γH2AX in T98G treated with TMZ and Cobi. **(C)** Calculation and visualization of synergy scores for drug combinations of Cobi and TMZ. **(D)** Representative images and quantification of cell apoptosis measured by flow cytometry (n = 3). *p*-values obtained by one-way ANOVA. **(E)** Immunoblot in GSCs treated with TMZ and clobetasol propionate (CP). **(F)** Cell viability assay with indicated concentrations of the combination of TMZ and CP in GSCs (n = 3). *P* values were obtained by one-way ANOVA. **(G-H)** Representative images and quantification of neurosphere formation in GSCs treated with TMZ and CP (n = 3). *P* values were obtained by one-way ANOVA. **(I)** Calculation and visualization of synergy scores for drug combinations of CP and TMZ. **(J)** Histological analysis of the liver, heart, and kidney of mice with indicated treatment. Scale bar: 50 µm. Error bars represent mean ± SD. Significant results are presented as, ns *P*>0.05, **P* < 0.05, ***P* < 0.01, ****P* < 0.001, *****P* < 0.0001.
