# Supplementary material for: CYP3A5 promotes glioblastoma stemness and chemoresistance through fine-tuning NAD+/NADH ratio
Source: J Exp Clin Cancer Res. 2025 Jan 3;44:3. doi: 10.1186/s13046-024-03254-x (PMC11697892; doi:10.1186/s13046-024-03254-x)
Supplement: Supplementary file 1 — Supplementary Material 1 [file 13046_2024_3254_MOESM1_ESM.docx]

Dear reviewers:

We would like to express our deepest appreciation for your time and effort in handling our manuscript. Please find our point-to-point responses to the reviewers’ comments as detailed below. We are hopeful that all of the additional data, analyses, and information we have provided satisfy the reviewers’ concerns.

**Reviewer #1:**

**Hu et al. identified a novel GBM treatment target, CYP3A5, which sustains GBM stemness and chemoresistance through modulating NAD+/NADH ratios. They provided useful information for readers interested in glioma. However, some concerns need to be solved before publication.**

**Overall Response:**
We thank the reviewer for recognizing our work and for the helpful and constructive comments, all of which have been addressed, as detailed below.

**Comment 1:
This manuscript suggests that CYP3A5 promotes GSCs by modulating NAD+/NADH redox balance. Since CYP3A5 belongs to the CYP3A family that has the same function of converting NADH to NAD+, do other members of the CYP3A family have a similar role in GBM progression?**

**Response 1:**

Thank you for your insightful comment regarding the potential roles of other members of the CYP3A family in glioblastoma stem cell (GSC) biology and GBM progression. We explore the expression pattern of three CYP3A family members, namely CYP3A5, CYP3A4, and CYP3A7, across normal brain tissue and low-grade glioma (LGG) and high-grade glioma (HGG), as shown in the following **letter Figure1**. Compared to CYP3A5, other members of the CYP3A family are either minimally expressed or exhibit significantly lower expression levels in glioma. These results indicate that, unlike CYP3A5, high-grade glioma does not exhibit an expression preference for other members of the CYP3A family. Therefore, we propose that other members of the CYP3A family might not have a similar role as CYP3A5 in GBM progression.

**Letter Figure 1**: The expression levels of CYP3A family members across normal brain tissue, low-grade glioma (LGG), and high-grade glioma (HGG). The gene expression was shown in log TPM from GTEx and TCGA GBMLGG dataset.

**Comment 2:**
**MGMT is a key player in the chemoresistance of gliomas, particularly in response to alkylating agents like TMZ. Does CYP3A5 inhibitor also augment TMZ-induced DNA damage in MGMT high-expressing GBM cells?**

**Response 2:**

We appreciate your insightful comment regarding the role of MGMT in GBM chemoresistance and its interaction with temozolomide (TMZ). In response to your question about whether CYP3A5 inhibitors also enhance TMZ-induced DNA damage in MGMT high-expressing GBM cells, we tested the impact of the CYP3A5 inhibitor on DNA damage in MGMT high-expressing T98G GBM cell line (**Shown in revised Figure S7B**). As shown in Figure S7B, TMZ alone does not induce observable DNA damage in MGMT high-expressing T98G GBM cells. However, the combination of TMZ with a CYP3A5 inhibitor caused a significant elevation of γH2AX, indicating CYP3A5 inhibitor also augments TMZ-induced DNA damage in MGMT high-expressing GBM cells.
**Comment 3:**

**CYP3A5 has been well-documented to facilitate widespread resistance to therapies in other cancer (PMID: 37679568 ). This manuscript provides a novel insight into how CYP3A5-mediated alteration of the activity of PARP promotes glioma chemoresistance. Thus, the roles of CYP3A5 in cancer resistance should be discussed accordingly.**

**Response 3:**

We appreciate the reviewer’s insightful comment regarding the role of CYP3A5 in facilitating resistance to cancer therapies. We acknowledge that CYP3A5 has been well-documented in various cancers, including its involvement in drug metabolism and resistance mechanisms (PMID: 37679568). Our manuscript aims to provide a novel insight into how CYP3A5-mediated alterations in PARP activity contribute specifically to glioma chemoresistance. To address the reviewer’s suggestion, we have expanded the discussion section to include a more comprehensive analysis of CYP3A5's roles in chemoresistance.

**Comment 4:**
**In addition to NAD+/NADH balance, the synthesis of NAD is also altered in glioblastoma. Some targets concerning NAD biogenesis have also been reported to affect GBM stemness, resistance, etc. Does CYP3A5 impact the expression of NAD synthesis-related genes, such as NAMPT, NAPRT, etc?**

**Response 4:**

Thank you for your insightful comment regarding the role of NAD metabolism in GBM and the potential influence of CYP3A5 on NAD synthesis-related genes such as NAMPT and NAPRT. As shown in the **letter Figure 2**, we found that after CYP3A5 KO, the expression of NAD synthesis-related genes, such as NAMPT and NAPRT, were slightly increased. Given the fact that CYP3A5 KO decreases the transition of NADH to NAD+, we reason that this decrease of NAD+ might activate the expression of NAD synthesis-related genes for feedback to restore the NAD+ level in cells. Again, this observation further verifies our conclusion that CYP3A5 KO reduces the NAD+ level in GSCs.

**Letter Figure 2**: The mRNA expression of NAMPT, and NAPRT in CYP3A5-KO GSCs conducted by RT-qPCR.

**Comment 5:**
**In the introduction, the latest research progress on the mechanism of TMZ resistance in glioma should be concentrated on.**

**Response 5:**

Thank you for your valuable feedback regarding the introduction. We appreciate your suggestion to focus more on the latest research progress concerning TMZ resistance in gliomas. And we have included important mechanisms of TMZ resistance research in the introduction part.

**Comment 6:**
**Page 6 lines 141-143 "This is attributed to the role of the mitochondrion in supporting tumor metabolic adaptation through energy production and biosynthetic intermediates." Please provide reference here.**
**Response 6:**

Thank you for your valuable comment regarding the need for a reference on our statement about the role of mitochondria in supporting tumor metabolic adaptation through energy production and biosynthetic intermediates. We appreciate your suggestion and have included the relevant reference to support this statement.

**Comment 7:**
**Figure 7D should be analyzed quantitively.**
**Response 7:**

Thank you for your valuable feedback regarding Figure 7D. We appreciate your suggestion to analyze this figure quantitatively. We agree that a quantitative analysis would enhance the clarity and impact of the results presented in Figure 7D. In the revised manuscript, We have included a detailed quantitative analysis of the data represented in this figure (**Shown in revised Figure 7D**).

**Reviewer #2**
**In their manuscript entitled "CYP3A5 promotes glioblastoma stemness and chemoresistance through fine-tuning NAD+/NADH ratio" Hu et. al. seek to understand how metabolic reprogramming contributes to the stem cell and tumor phenotype of gliomas. Using a combination of bioinformatics, molecular, cellular and pharmacological approaches the authors present a well-balanced study addressing this hypothesis. Using ML approaches the investigators constructed a stemness score (GScore) used to stratify patients and identify targetable genes enriched in these patient populations. Using this approach, the authors identified CYP3A5 as high-priority candidate and very rigorously show this gene controls the stem cell and tumor phenotype of GBM. The study is well-controlled and the figures are presented clearly. The authors did a great job using multiple complementary approaches to test their hypothesis and validate the associations produced by the bioinformatics analyses. However, there are lapses in the writing and presentation of the observations and results that need to be addressed, as well as minor conceptual concerns. Overall, this is very nice study that should appeal to the general audience and those studying GSCs in particular. Job well done!**
**Overall Response:**
We appreciate the reviewer for acknowledging our work and for the constructive comments, all of which we have addressed in detail below.

**Comment 1:**
*** There is an abundance of unfinished thoughts. For instance, in the abstract the authors state "NAD(H) emerges as a crucial factor influencing GSCs", what aspect of GSC biology or the cancer stem cell phenotype is being 'influenced' is very unclear. The text needs to be proofread to address these concerns.**

**Response 1:**

Thank you for your valuable feedback regarding our manuscript. We acknowledge that the statement in the abstract regarding NAD(H) as a crucial factor influencing glioblastoma stem cells (GSCs) was not sufficiently clear. In the revised manuscript, we have elaborated on how NAD(H) affects specific aspects of GSC biology, such as tumor initiation, mitochondrial function, and DNA damage repair. This clarification is now included in both the abstract and the relevant sections of the main text.

We also have thoroughly proofread the manuscript to address any unfinished thoughts and enhance overall clarity. We have made revisions to ensure that all statements are well-supported and clearly articulated throughout the text.

**Comment 2:**
*** The authors should consider presenting the paragraph starting with "Metabolic reprogramming is a hallmark of tumors…" in the introduction prior to the one beginning with "Nicotinamide adenine dinucleotide (NAD) is a crucial small molecular…".**

**Response 2:**

Thank you for your insightful suggestion regarding the organization of the introduction. We agree that presenting the paragraph beginning with "Metabolic reprogramming is a hallmark of tumors..." prior to the one starting with "Nicotinamide adenine dinucleotide (NAD) is a crucial small molecule..." enhances the logical flow of the manuscript. We have made this adjustment in the revised version to improve clarity and coherence.

**Comment 3:**
*** Information of antibodies used for IF, ChIP, IHC seems to be missing. Please clarify in table S1 which ones were used for these assays or provide the missing information.**

**Response 3:**

Thank you for your helpful comment regarding the information on antibodies used for IF, ChIP, and IHC. We apologize for the oversight and have updated Table S1 to include detailed information about the antibodies used in these assays (**shown in revised Table S1**). This includes the specific antibodies, their sources, any relevant dilution factors, and applications.

**Comment 4:**
*** For reproducibility purposes, please provide tables with the genes used to generate the different signatures presented in the study, including the GScore, or provide or provide identifiers if retrieved from GSEA or other repository.**

**Response 4:**

Thank you for your feedback. We have provided all relevant GSEA pathway gene sets used in this study in **new Table S5**, including the top gene sets mentioned in Figure 1 and mitochondrial-related gene sets in Figure 5. Additionally, we would like to clarify that genes and relevant coefficients for GScore formulation have been provided in the **revised Table S4**.

**Comment 5:**
*** Fig 1B is very complex and has a lot of information, however, the authors only describe the this results in one sentence. It is very unclear what the purpose of this analysis was and the information presented in this panel is a lot to unpack for the average reader. Please provide an interpretation of the data being presented highlighting the salient features of the analysis in the context of this study.**

**Response 5:**

Thank you for your thoughtful feedback regarding Figure 1B. We appreciate your recognition of its informative nature. The primary aim of this analysis is to illustrate the general landscape of GScore in relation to various clinical and molecular factors among glioma patients. We believe this approach provides readers with a straightforward presentation of how GScore correlates with key glioma characteristics. For instance, as GScore increases, glioma patients tend to present with higher WHO grades and shorter overall survival rates. Additionally, we can see from Fig.1B that patients with elevated GScore are more likely to be IDH wildtype and exhibit MGMT promoter unmethylation. These all indicate that higher GScore is associated with worse glioma clinical outcomes.

In response to your comments, we have expanded the description of Figure 1B in the revised manuscript to offer a more detailed and clear interpretation of these results for average readers. Thank you once again for your valuable insights!

**Comment 6:**
*** Similar to the comment above, the description about the mDNAsi is very vague. Why was this analysis included, what does this information mean in the context of the main hypothesis being tested, why is this information worth presenting? The authors should keep in mind that just because an analysis was performed and results were generated, they don't always need to be presented. The authors urged to edit and only present the results and analyses directly relating to the hypothesis being tested.**

**Response 6:**

Thank you for your valuable feedback regarding the mDNAsi analysis in our manuscript. As we originally included in the method part of the manuscript, mDNAsi calculation is the intermediate step for GScore construction. And, the primary purpose of the presentation about mDNAsi in the original Fig.1C is to delineate that mDNAsi-derived GScore well reflects the feature of mDNAsi. Nonetheless, we realize that it is unnecessary to present these results due to these results are not directly related to the hypothesis being tested in this part. Moreover, in light of our primary objective to illustrate the clinical implications and stemness relevance of GScore in Figure 1, we have decided to remove the mDNAsi results from this figure (**shown in revised Fig. 1**). We hope this adjustment will allow us to maintain a focused presentation that directly supports our main hypothesis.

We also have thoroughly proofread the manuscript to reconsider those results and analyses not directly relating to the hypothesis being tested. Therefore, the original Fig. 1E, Fig. S3I, and Fig S3K have also been decided to not present (**shown in revised Fig. 1 and Fig. S3**).

**Comment 7:**
* The statement "clinical outcomes of both glioma and GBM patients" is misleading. GBM is a type of glioma, are the authors trying to make a distinction between IDH wild-type and mutant gliomas? Please clarify

**Response 7:**

We appreciate the reviewer’s insightful comment regarding this statement. We would like to clarify that the primary aim of this statement is to convey that the GScore can predict clinical outcomes for all glioma patients, including both low-grade and high-grade gliomas. Additionally, the GScore is also effective in predicting clinical outcomes specifically for GBM patients.

Nonetheless, we recognize that GBM is indeed a type of glioma, and our original statement might have caused confusion among readers. To address this concern, we have revised the statement to: '**Altogether, these results indicate that the glioma stemness index-derived GScore predicts clinical outcomes for glioma patients.**' We hope this revision provides greater clarity and accurately reflects our findings.

**Comment 8:**
* The statemen in line 457 - Strikingly, in contrast to the low-score group…- is very misleading. It is not striking at all that these signatures are enriched in these patient subsets since the main criteria to separate them was a "stemness" score. If anything, these correlations are consistent with the inclusion criteria. This need to be reworded.

**Response 8:**

Thank you for your valuable feedback regarding the statement in line 457. The use of 'strikingly' was intended to highlight the notable differences observed in stemness signature enrichment between the low-score group and high-score subsets of glioma patients. However, We understand your point that these findings may seem expected given the inclusion criteria based on 'stemness' scores.

In light of your feedback, we have reworded the statement to reflect this context better. The revised line now reads: “**As expected, in contrast to the low-score group, …**”. We hope this revision accurately conveys our findings without implying any unexpected results.

**Comment 9:**
* It is unclear what is the relevance of the immunosuppressive score. Although an immunosuppressive environment is a feature of glioma in general, this study does not look at metabolic reprogramming in this context. Please clarify the relevance of this information.

**Response 9:**

Thank you for your thoughtful feedback regarding the relevance of the immunosuppressive score in our study. The main purpose of including immunosuppressive scores is to expand the clinical applicability of the GScore in light of the close relationship between tumor stemness and tumor suppressive immunity. Specifically, we aim to demonstrate that the GScore not only serves as a predictor of glioma stemness features and clinical outcomes but also offers insights into the immune microenvironment status of glioma patients. By broadening the clinical applicability of the GScore, we believe it can provide a more comprehensive framework for patient stratification and inform precise therapeutic strategies for both clinical practice and trials.

In response to your feedback, we have revised our manuscript to elaborate more explicitly on this point and discuss more about its anticipated implications. Thank you once again for your valuable comments.

**Comment 10:**
* It is unclear how the drug screen was performed in Fig. 2. Please provide information about the format of the screen, endpoints used, cell lines used for the screen, etc.

**Response 10:**

Thank you for your valuable feedback regarding the drug target screening methodology presented in Fig. 2. To identify druggable target genes that may have potential therapeutic implications for patients with high stemness status, a total of 2249 drug-target genes were included for selection. These drug-target genes were derived after duplication removal of more than 6,000 compounds, many of which have been FDA-approved. These data were retrieved from the Drug Repurposing Hub created by Broad Institute, which aims to take thousands of drugs already approved to safely treat disease, as well as other compounds that have been studied as potential drugs, and find new uses for these old medicines.

Then, among these drug-target genes, glioma stemness-related genes were identified with gene expression levels positively correlated to GScores Next, we calculated the GScore for each glioma cell line from the CCLE project and retrieved the Chronos score of these cell lines from the DepMap dataset. Chronos scores represent the importance of a gene of interest for a given cell line through cell depletion assay, a lower Chronos score of the gene denotes a more essential role in cell fitness. Therefore, glioma-dependent genes were identified with Chronos score inversely associated with the GScore. Finally, four potential therapeutic drug targets —CKMT2, CYP3A5, CLCN2, and PIEZO1—were then derived by combining both analyses above Among four candidates, CYP3A5 expression indicates the worst prognostic outcomes by the single covariate Cox proportional hazards model in glioma patients. Moreover, we quired the Cmap database to assess the perturbation effect of gene depletion on tumor stemness status. Connectivity scores were then obtained by this analysis; a lower connectivity score signifies a stronger ability of a certain target depletion in disrupting the tumor's ability to maintain stem-like properties in this context. In glioma cell line U251MG, we found that CYP3A5 depletion had the lowest normalized connectivity score, suggesting the strongest ability to reverse the high GScores signature for glioma. Taken together, CYP3A5 may be the potential druggable target for glioma patients with high stemness status.

We have revised our manuscript to elaborate more explicitly on the drug target screening process.

**Comment 11:**
*** The authors need to be considerate to the average reader and provide all the necessary information for the audience to be able to digest the findings properly. What is a "Chronos score", what features are incorporated into the score that address questions relevant to the study.**

**Response 11:**

Thank you for your insightful feedback regarding the clarity of our manuscript. Chronos is an algorithm for inferring gene fitness effects from CRISPR knockout experiments, which represents the importance of a gene of interest for a given cell line. Gene with a lower Chronos score represents that it has a more essential role in supporting cell growth and survival. In the context of cancer cells, genes that have a lower Chronos score are more likely to be exploited to target cancer cell growth. In this study, we retrieved the gene Chronos scores of glioma cell lines from the Dependency Map (DepMap) portal to screen the target genes that might be essential for glioma growth and survival. In the revised manuscript, we have included a clear definition of the Chronos score.

Moreover, we have reviewed any other technical language and unfinished thoughts, such as mDNAsi, the connectivity score, and JASPAR binding scores, throughout the manuscript to ensure that it is accessible to a broader audience, particularly those who may not have a specialized background in computational biology and technology.

**Comment 12:**

* There is a seeming discrepancy between the GScore analysis presented in Fig.1 and the validation presented at the end of Fig. 2. All the analyses in Fig. 1 points at a mesenchymal transition correlated with a more aggressive stem cell and tumor phenotype in gliomas, consistent with the current dogma in the field. However, the validation highlights the co-expression of CYP3A5 with Sox2 and CD133, stemness genes associated with the proneural state which, coincidentally, the authors show is depleted in their analysis (Fig. 1K). Why was CD133 and Sox2 used as markers and not mesenchymal markers (e.g. CD44). This aspect requires substantial attention.

**Response 12:**

We thank the reviewer for their critical observation regarding the potential discrepancy between the GScore analysis in Fig.1 and the CYP3A5 validation presented in Fig. 2, and marker selection issues.

In Fig. 1, we find that the high GScore group enriches gene sets referring to less differentiated neural stem cells, such as radial Gila cells, oligodendrocyte precursor cells (OPC), neural stem cells (NSC), etc. In contrast, the low GScore group enriches gene sets pertaining to more mature brain cells (e.g., neurons, microglia ). Therefore, we believe that GScore is a promising indicator for the stemness status of GBM. Next, we identify a potential target CYP3A5 based on GScore for GBM patients with high stemness status.

Indeed, as indicated in Fig. 1, GScore is higher in mesenchymal GBM than in proneural GBM, yet we did not find significant differences in levels of CYP3A5 between mesenchymal and proneural GBM patients (**shown in new Figure S4A**). Moreover, as indicated in original Fig. S3H, CYP3A5 expression is positively correlated to most stemness-related genes including proneural and mesenchymal GSC markers. These lead us to propose that CYP3A5 may play a consistent role across both proneural and mesenchymal GSCs (as discussed more below).

In Fig. 2, CD133 and SOX2 were selected due to their established significance in GSC biology and their widespread use for GSCs identity (PMID: 15549107, PMID 18948646). However, there are also other studies pointing to the co-existence of two different types of GSCs (proneural and mesenchymal GSCs) within different regions of a single GBM patient (PMID: 29035367, PMID: 23650391). Since CD133 and SOX2 are typically markers for proneural GSCs, we recognize that exploring the role of CYP3A5 in mesenchymal GSCs for a more general implication for GBM stemness is also important. To this end, we further investigated the expression of CYP3A5 in GSCs that exhibit mesenchymal markers (CD44, BMI1). Similar to the aforementioned proneural markers, CYP3A5 is also highly expressed in mesenchymal GSCs, supported by both single-cell RNA sequencing data and co-IF analysis of GBM samples (**shown in new Figures S4B and S4C**). Moreover, knocking down CYP3A5 expression in the patient-derived mesenchymal GSC line BNI-20-1-S also resulted in a reduction of self-renewal capability (**shown in new Figures S4D and S4E**).

Collectively, these results demonstrate that CYP3A5 upregulated in GSCs (both in proneural and mesenchymal GSCs) is associated with GBM progression and stemness, supporting CYP3A5 as a potential GBM therapeutic target.
